# Herb pair Danggui-Honghua: mechanisms underlying blood stasis syndrome by system pharmacology approach

**DOI:** 10.1038/srep40318

**Published:** 2017-01-11

**Authors:** Shi-Jun Yue, Lan-Ting Xin, Ya-Chu Fan, Shu-Jiao Li, Yu-Ping Tang, Jin-Ao Duan, Hua-Shi Guan, Chang-Yun Wang

**Affiliations:** 1 Key Laboratory of Marine Drugs, The Ministry of Education of China, School of Medicine and Pharmacy, Ocean University of China, Qingdao 266003, P. R. China; 2 Laboratory for Marine Drugs and Bioproducts, Qingdao National Laboratory for Marine Science and Technology, Qingdao 266071, P. R. China; 3 Jiangsu Key Laboratory for High Technology Research of TCM Formulae, Nanjing University of Chinese Medicine, Nanjing 210023, P. R. China

## Abstract

Herb pair Danggui-Honghua has been frequently used for treatment of blood stasis syndrome (BSS) in China, one of the most common clinical pathological syndromes in traditional Chinese medicine (TCM). However, its therapeutic mechanism has not been clearly elucidated. In the present study, a feasible system pharmacology model based on chemical, pharmacokinetic and pharmacological data was developed via network construction approach to clarify the mechanisms of this herb pair. Thirty-one active ingredients of Danggui-Honghua possessing favorable pharmacokinetic profiles and biological activities were selected, interacting with 42 BSS-related targets to provide potential synergistic therapeutic actions. Systematic analysis of the constructed networks revealed that these targets such as HMOX1, NOS2, NOS3, HIF1A and PTGS2 were mainly involved in TNF signaling pathway, HIF-1 signaling pathway, estrogen signaling pathway and neurotrophin signaling pathway. The contribution index of every active ingredient also indicated six compounds, including hydroxysafflor yellow A, safflor yellow A, safflor yellow B, *Z*-ligustilide, ferulic acid, and *Z-*butylidenephthalide, as the principal components of this herb pair. These results successfully explained the polypharmcological mechanisms underlying the efficiency of Danggui-Honghua for BSS treatment, and also probed into the potential novel therapeutic strategies for BSS in TCM.

With the ever-increasing acceptance of combination therapy and the prevalence of chronic diseases in the world, the use of traditional Chinese medicine (TCM) has become an emerging trend^1^. A TCM prescription usually contains numerous ingredients synergistically and holistically acting on the diseases. Herb pairs, the simplest form and the centralized representative of Chinese herbal compatibility, intrinsically convey the basic idea of TCM prescriptions^2^. Danggui and Honghua combined as an herb pair have been frequently used in TCM prescriptions, which could be ascended to an ancient and classical formula Danggui-Honghua Decoction. Danggui, the radix of *Angelica sinensis* (Oliv.) Diels, has been used for thousands of years in the East Asia and was firstly recorded in a classical masterpiece of TCM *Shennong Bencao Jing* (200–300 A.D., Han Dynasty). Danggui has also been called “female ginseng”, predominantly renowned for treatment of intractable gynecological disorders^3^. Honghua, the dried florets of *Carthamus tinctorius* L., has been used extensively in TCM to treat stroke, coronary heart disease and angina pectoris. In the *Compendium of Materia Medica*, Honghua was described as being able to invigorate the circulation of blood^4^. Other classical formulae, such as Tao-Hong-Si-Wu Decoction, Xue-Fu-Zhu-Yu Decoction, and Bu-Yang-Huan-Wu Decoction, also contain these two herbs^5^.

Danggui-Honghua is commonly used for treating clinical blood stasis, which is described as slowing or pooling of the blood due to disruption of heart Qi of TCM^6^. Ancestors and modern scholars considered that “sorrow nu” of the seven emotions and “cold evil” of the six evils were the primary causes of acute blood stasis^7^. Modern pathology shows that blood stasis is generally manifested by cardio-cerebrovascular diseases such as myocardial infraction, coronary heart disease, and high blood pressure resulting from hematological disorders including hemorrhage, congestion, thrombosis, and local ischemia^6^,^8^. Although well-practiced in clinical medicine, the mechanisms of how Danggui-Honghua exerts the therapeutic effects on blood stasis syndrome (BSS) remain elusive.

System biology is now considered as a holistic and efficient tool to study the role of TCM^1^. Combined with pharmacology and pharmacodynamics, system biology has given birth to a promising subject, i.e., system pharmacology^9^. On this basis, an integrated mode combining pharmacokinetics prediction and network pharmacology techniques has been developed and successfully applied to interpret the mechanisms of several Chinese herbal medicines and formulae at molecular network level^10^,^11^. In the present study, the mechanisms of herb pair Danggui-Honghua in treating BSS were investigated by the system pharmacology model based on chemical, pharmacokinetic and pharmacological data together with contribution index analysis.

## Results

**Figure 1:**
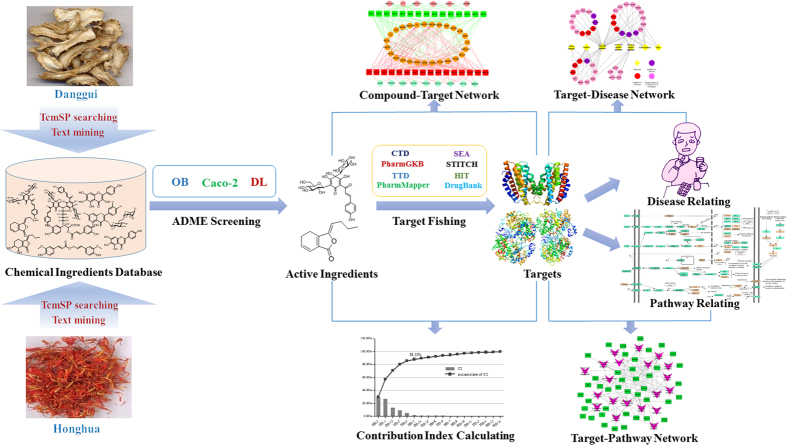
The whole framework based on an integration strategy of system pharmacology.

The molecular mechanisms of the herb pair Danggui-Honghua against BSS were investigated by a network construction approach with system pharmacology model based on chemical, pharmacokinetic and pharmacological data (Fig. 1). All of the ingredients from this herb pair were explored to build a compound library. Next, the oral bioavailability (OB), Caco-2 cell permeability and drug-likeness (DL) of the ingredients were collected and calculated to screen the potential active compounds. Then the potential targets and their corresponding diseases and pathways of the active compounds were data-mined from literature and public database/software sources. Subsequently, the acquired pharmacological data were integrated into the compound-target (C-T), target-disease (T-D), and target-pathway (T-P) networks, respectively. Finally, a contribution index of every active ingredient based on network based efficacy weighted by literature was calculated. Based on the above analyses, the underlying mechanisms of Danggui-Honghua for treating BSS were explored.

### Ingredient comparisons in Danggui and Honghua

The ingredients in Danggui and Honghua were retrieved from Traditional Chinese Medicine Systems Pharmacology Database and Analysis Platform (TcmSP™, http://sm.nwsuaf.edu.cn/lsp/tcmsp.php)^12^ and were manually supplemented. The main components of Danggui are phthalides and organic acids^13^, whereas the major ingredients in Honghua are quinochalcone *C*-glycosides and flavonoid glycosides possessing one or more glucose units^4^. Since those glycosides in this herb pair might be deglycosylated by the glycosidase in the intestinal tract, 11 aglycones were also incorporated into the compound library labeled by_qt. Thus, a total of 347 ingredients were retrieved for Danggui (143) and Honghua (204). The detailed information about these molecules was provided in Supplementary Table S1.

**Figure 2:**
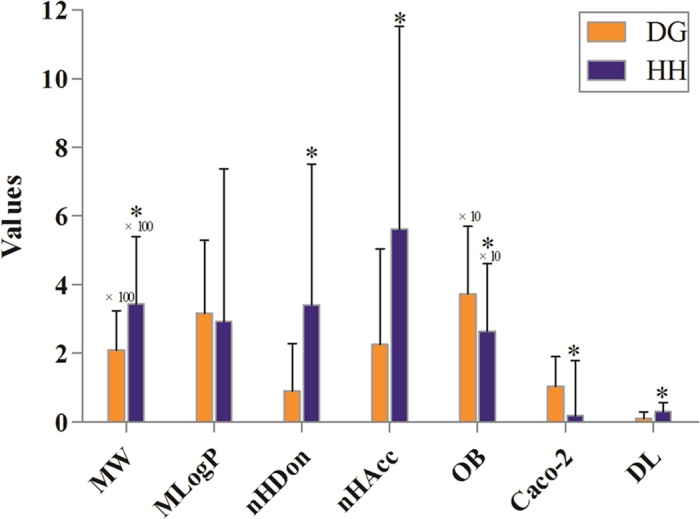
The molecular diversity of all ingredients from Danggui and Honghua. Molecular properties consist of molecular weight (MW), Moriguchi octanol-water partition coeff. log *P* (MLogP), number of donor atoms for H-bonds (nHDon), number of acceptor atoms for H-bonds (nHAcc), oral bioavailability (OB), Caco-2 permeability (Caco-2) and drug-likeness (DL). *P < 0.05 by two tailed t-test (vs. Danggui).

To investigate the molecular diversity of the ingredients from Danggui and Honghua, ingredient comparisons were conducted based on seven significant properties, including the molecular weight (MW), Moriguchi octanol–water partition coeff. (LogP) (MLogP), the number of donor atoms for H-bonds (nHDon), the number of acceptor atoms for H-bonds (nHAcc), OB, Caco-2 and DL (Fig. 2). (1) From the MW, the distinct average numbers of MWs of the constituents from Danggui (208.71) and Honghua (343.94) were observed. By two tailed *t*-test, the MWs of the individual compounds from these two herbs were significantly different (*P* = 7.36E–13). (2) The MLogP values of the constituents from Danggui (3.16) and Honghua (2.93) were similar and displayed no significant difference (*P* = 0.62), indicating that the majority of the ingredients in both herbs were hydrotropic. (3) The average nHDon number of Danggui constituents (0.90) was significantly lower than that of Honghua constituents (3.41) (*P* = 1.23E–11). (4) The average nHAcc number of Danggui constituents (2.26) was also significantly lower than that of Honghua constituents (5.62) (*P* = 8.84E–10). (5) Compared with Honghua constituents (average OB value = 26.48), Danggui constituents possessed higher average OB value of 37.28 (*P* = 3.50E–7). (6) For permeability, the average Caco-2 value of Danggui constituents (1.05) was significantly higher than that of Honghua constituents (0.18) (*P* = 8.99E–9). (7) Whereas for DL analysis, unlike OB and Caco-2 parameters, Danggui constituents exhibited lower average DL index (0.11), which is significantly different from that of Honghua (0.30) (*P* = 2.44E–12).

The above analysis suggested that the constituents of Danggui and Honghua were diverse but the majority of them satisfied the Lipinski’s rule of five. Apart from the hydrotropic property, Danggui was significantly different from Honghua in other properties of the chemical components. These differences are not surprising due to the distinct chemo-physical properties of the ingredients from these two herbs. The above results also showed that the constituents from Danggui have better pharmacokinetic properties (OB and Caco-2), whereas the ingredients from Honghua possess better drug-likeness (DL). From a chemotaxonomic point of view, phthalides from Danggui and quinochalcone *C*-glycosides from Honghua have only been found in the families Apiaceae and Asteraceae, respectively. Although these major ingredients between Danggui and Honghua are obviously different, the two herbs exhibit the identical blood-activating and stasis-dissolving effects and the warm nature of TCM, which may also clarify why Danggui-Honghua could produce synergistic and complementary effects.

### Active ingredients in Danggui-Honghua

Although a single herb or TCM formula usually contains a considerable number of bioactive components, maybe only a few with desirable pharmacodynamic and pharmacokinetic properties are responsible for its therapeutic effects. In the present work, three crucial ADME (absorption, distribution, metabolism, and excretion) parameters, including OB, Caco-2, and DL, were employed to screen most of the active compounds from Danggui-Honghua. A few active compounds that do not meet all of these three criteria were also selected for their high amounts and high bioactivities. Consequently, a total of 31 active compounds were selected from the 347 compounds of this herb pair (Table 1).

### Active ingredients from Danggui

By ADME screening, 15 out of 143 ingredients with excellent pharmacological effects were extracted from Danggui, and the majority of them possess satisfactory pharmacokinetic profiles. For instance, caffeic acid (DG-14, OB = 54.97%, Caco-2 = 0.21 and DL = 0.19) has antioxidant, anti-inflammatory, antimutagenic, antibacterial and anti-carcinogenic effects^14^. Likewise, *β*-sitosterol (DG-15, OB = 36.91%, Caco-2 = 1.33 and DL = 0.75) exhibits potent anti-inflammatory and antipyretic activities^15^. It should be point out that phthalides, the representative ingredients in Danggui, show low DL values, but they exhibit potent antifungal, antibacterial, anti-inflammatory, and antioxidant activities^16^. Similarly, although phenolic constituents hold low DL values, they also exhibit remarkable pharmacological effects^17^,^18^. Specifically, *Z*-ligustilide (DG-1) and ferulic acid (DG-12) have been chosen as the marker components for quality control of Danggui in *Chinese Pharmacopoeia*^19^. In view of the facts mentioned above, phthalides and phenolic constituents were also deemed as the active ingredients for further analysis. Furthermore, the contents of the selected constituents in Danggui were also considered. Through a wide-scale text mining of Google Scholar, the total content of ferulic acid, *Z-*butylidenephthalide (DG-5), senkyunolide A (DG-2), senkyunolide I (DG-3), and *Z*-ligustilide was found to be nearly 43 mg/g^20^, and nicotinic acid (DG-8) was up to 0.198 mg/g^21^. Noteworthy, coniferyl ferulate (DG-9) is also abundant in Danggui and exhibits multiple biological activities such as antioxidant, vasodilating and antibacterial effects^18^. Nodakenin (DG-11) has been reported to possess neuroprotective, anti-inflammatory, antibacterial, and memory-improving effects^22^. Based on the above considerations, it was reasonable to believe that fifteen compounds could be listed as potential active ingredients for Danggui (Table 1).

### Active ingredients from Honghua

In Honghua, only 16 ingredients passed through the strict filtering criteria, and most of them exhibit potent pharmacological activities. For examples, quercetin (HH-9, OB = 46.43%, Caco-2 = 0.05 and DL = 0.28) presents anti-inflammatory, anti-proliferative, and hepatoprotective activities^23^; luteolin (HH-15, OB = 36.16%, Caco-2 = 0.19 and DL = 0.25) shows antioxidant, anti-inflammatory and anti-allergic effects^24^. Surprisingly, quinochalcone *C*-glycosides, the main active and characteristic compounds in Honghua, exhibit low OB and Caco-2 values. However, HSYA (HH-3, OB = 4.77%, Caco-2 = −2.77 and DL = 0.68) has been chosen for the quality control of Honghua in *Chinese Pharmacopoeia*^25^ and has been developed into the intravenous injection in China to treat cardiac-cerebral vascular ailments with good clinical effects^4^. Safflor yellow B (HH-5, OB = 12.00%, Caco-2 = −2.40 and DL = 0.67) has also shown potent neuroprotective and anti-oxidative abilities on cerebral ischemic injury^26^. Thus, quinochalcone *C*-glycosides were also selected for targeting. Notably, scutellarin (HH-12, OB = 2.64%, Caco-2 = −1.08 and DL = 0.79) has been used in tablet and injection forms in China since 1984 for treatment of acute cerebral infarction and paralysis induced by hypertension, cerebral thrombosis, and cerebral hemorrhage^27^. Scutellarein (HH-13), the aglycone of scutellarin which is a candidate drug for cardio-cerebrovascular diseases was also selected for our further analysis. In addition, it is necessary to incorporate nicotiflorin (HH-8, OB = 3.64%, Caco-2 = −1.77 and DL = 0.73) into further investigation for that this compound has potent neuroprotective, analgesic, anti-hypertensive and anti-anaphylactic effects^28^.

### Target proteins of Danggui-Honghua

Searching for the targets of candidate drugs solely by the experimental approaches is overspending, labor-intensive, and time-consuming. In the present work, an integrated *in silico* approach was introduced to identify the target proteins for the active ingredients of Danggui-Honghua. Predictive models were used including Similarity Ensemble Approach (SEA, http://sea.bkslab.org/)^29^, STITCH (http://stitch.embl.de/)^30^ and PharmMapper sever (http://59.78.96.61/pharmmapper)^31^, and databases were mined including Herbal Ingredients’ Targets database (HIT, http://lifecenter.sgst.cn/hit/)^32^, Therapeutic Targets Database (TTD, http://bidd.nus.edu.sg/group/ttd/)^33^, DrugBank (http://www.drugbank.ca/) and Google Scholar. Finally, 42 targets related to BSS were determined, interacting with the selected 31 active ingredients of this herb pair (Table 2).

### Target proteins of Danggui

Thirty-three targets were identified for 15 active ingredients of Danggui with 108 interactions. Multiple therapeutic targets concerning BSS were mediated by the active ingredients of Danggui, such as ESR1, ESR2, PTGS1, PTGS2, JUN, ICAM1, NOS2, NOS3, and MAPK1. Most of these targets are involved in vascular and central neural systems. For instances, ESR1 and ESR2 mediate the vascular system to promote the functional recovery of vascular injury and provide neuroprotective effects in central neural system^34^; PTGS1 and PTGS2 contribute to atherosclerosis and thrombosis by regulating the production of eicosanoids that modulate physiological processes in the vessel wall^35^; JUN modulates smooth muscle cell proliferation in response to vascular angioplasty^36^; additionally, ICAM1 mediates the adhesion of neutrophils and monocytes to vascular endothelium^37^. Specifically, *Z*-ligustilide, senkyunolide I, ferulic acid and coniferyl ferulate may mediate NOS2 or NOS3 to increase the nitric oxide biosynthesis, thereby possibly exerting blood-vessel dilation, neuronal signal transmission, coordination of heart rhythm and regulation of cellular respiration activities^38^. Vanillin may interact with four potential targets including PTGS2, JUN, MMP9 and MAPK1 which are also relevant to nervous system and vascular diseases^39^.

Except for vanillin, nicotinic acid and *β*-sitosterol, other active ingredients from Danggui also interacted with the targets related to inflammation, abdominal pain, dysmenorrhea, embolism, thrombosis and ischemia. Six potential targets including F2, F7, F10, F11, TBXA2R and MAPK14 are relevant to disorders of thrombosis, which could clarify why Danggui exhibits strong anticoagulant function^16^. Additionally, there are also several targets such as PPARG, SOD1, RELA and GSK3B are involved in the ischemic and inflammation processes. It should be pointed out that some major targets, such as PTGS2, NOS3 and CHRM2, are also closely concerned with the pain-related diseases, which may contribute to the blood-activating and stasis-dissolving effects of Danggui.

### Target proteins of Honghua

For Honghua, by target fishing, 16 active ingredients were validated to bind with 36 target proteins related to BSS. There are also plenty targets mediated by the active ingredients from Honghua that are involved in vascular and central neural systems. For example, HSYA may have the potential to act on 18 targets including HIF1A, VEGFA, HMOX1, PTGS2, CASP3, CASP9, AGTR1, PTAFR, and GSK3B. Actually, HSYA has been identified as an inhibitor of HIF1A, which might contribute to its therapeutic application in vascular diseases^40^. Beyond that, it also has a strong antagonistic effect on the PTAFR protein, which may explain its function in both inflammatory and neuropathic pain responses^41^. Analogously, scutellarin exhibits strong neuroprotective effect because it may interact with STAT1, NOS2, NOS3, VEGFA and FGFR1 simultaneously^42^.

Inflammatory response is of enormous significance in BSS. Six active compounds of Honghua, including 6-hydroxykaempferol (HH-6), eriodictyol (HH-11), rosmarinic acid (HH-16), kaempferol, quercetin, and luteolin, may have interactions with PPARG which is expected to control inflammation associated with gut, myocardial, lung and cerebral ischemia^43^. Other five active ingredients, HSYA, rutin (HH-10), safflor yellow A (HH-2), safflor yellow B and eriodictyol, were identified to interact with PTGS1, which is a potential target for the next generation of anti-inflammatory drugs^44^.

Of note, we have implemented molecular docking combined with the *in vitro* experiments to explore the structure-activity relationships of the active ingredients among Si-Wu-Tang series containing the herb pair Danggui-Honghua^45^,^46^. Flavonoids including quercetin (IC_50_ = 0.035 mM), luteolin (IC_50_ = 0.052 mM), kaempferol (IC_50_ = 0.109 mM), and acacetin (IC_50_ = 0.140 mM) showed F2 inhibition activity *in vitro*. By the software of Molegro Virtual Docker (http://www.molegro.com), quercetin (−89.72 kJ/mol) exhibited high average MolDock score value and interacted with the active site residues Ala230, His79, Lys88, Gly258, Ser256, and Trp86 of F2^45^. It was also found that ferulic acid (60.45%), quercetin (70.00%), kaempferol (45.15%), HSYA (45.28%), rutin (90.83%), and scutellarin (53.46%) in 20 μg/mL showed higher estrogenic activity *in vitro* than caffeic acid (24.73%) in 20 μg/mL. And ferulic acid (−72.8 kJ/mol), quercetin (−76.3 kJ/mol) and rutin (−87.2 kJ/mol) exhibited high average MolDock score values and strong binding affinity to ESR1^46^. It could be concluded that the predicted targets were in agreement with our previous experiments. Therefore, the integrated *in silico* approach should be feasible and convincible to explore the compound-target interactions of Danggui-Honghua.

### Target and contribution index analysis to decipher the combination rule of Danggui-Honghua

**Figure 3:**
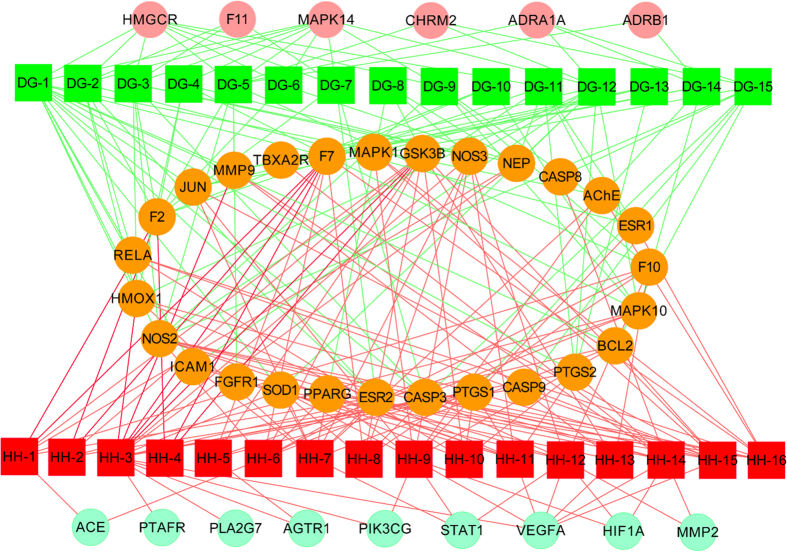
Compound-target network of Danggui-Honghua. Thirty-one active compounds (HH-/DG-, rectangle) map 42 potential protein targets (short name referred in Table 2, circle). The 15 bright green rectangles are active compounds from Danggui and the 16 red ones represent those from Honghua. The 27 orange circles are the proteins targeted by those compounds screened out of both herbs. The 6 dark pink circles on the upper side are the potential proteins hit by the compounds of Danggui and 9 tea green ones on the bottom side are the potential proteins only targeted by the compounds of Honghua.

To facilitate the visualization and interpretation of the complex relationships between all active ingredients of Danggui-Honghua and their targets, a bipartite graph of C-T network was constructed (Fig. 3). Two characteristics of Danggui-Honghua were observed from network analysis: (1) the promiscuous properties of its active ingredients and (2) the existence of highly interconnected compounds. The average number of potential targets per active ingredient was 6.5. And all active ingredients in this herb pair were potential multiple-kinase inhibitors or activators. Amongst them, those ones with high interconnection degrees were responsible for the high interconnectedness of the C-T network, especially HSYA (degree = 18), *Z*-ligustilide (degree = 15), quercetin (degree = 12), luteolin (degree = 11), ferulic acid (degree = 11) and *Z-*butylidenephthalide (degree = 10). From the topological features of this network and the functional properties of the proteins (Supplementary Table S2 and Fig. S1A), there were different modes of actions between compounds and targets in Danggui-Honghua. As shown in the C-T network (Fig. 3), the efficacy of this herb pair not only concentrated on modulating the crucial targets involving in the vascular and central neural systems (ESR1, ICAM1, HMOX1 and NOS3), but also, more essentially, focused on the regulation of the other proteins mediating inflammation, thrombosis, ischemia, dysmenorrhea and abdominal pain (F7, TBXA2R, PTGS2, CHRM2, and NOS2) to relieve the pathological changes and prolong the efficient curing process. For example, in animal models, increased expression of HMOX1 has been shown to protect tissues and cells against ischemia-reperfusion injury, oxidative stress, inflammation and hypoxia-induced vascular stasis^47^. The protein HMOX1 was found to have interactions with 9 ingredients, and senkyunolide A, kaempferol and eriodictyol synergistically increase HMOX1 expression^48^,^49^,^50^. In addition, the neurotoxic proinflammatory mediator PTGS2 was also connected with several active ingredients. Especially, HSYA, *Z*-ligustilide and ferulic acid were confirmed to enhance anti-inflammatory effects via significantly attenuatingthe expression level of PTGS2^4^,^51^,^52^.

**Figure 4:**
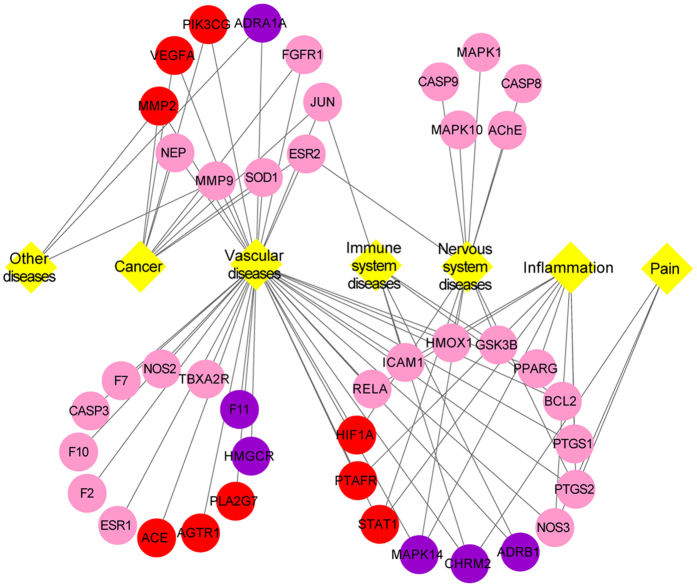
Target-disease network of Danggui-Honghua. The 6 purple circles and 9 red circles represent the targets of Danggui and Honghua, respectively. The 27 pink circles represent the targets shared by Danggui and Honghua. The 7 yellow diamonds represent the disorders related to those 42 targets.

The T-D network was further constructed based on all the targets and their corresponding diseases. As shown in the graphical network (Fig. 4), both Danggui and Honghua could regulate the proteins related to vascular and central neural system diseases as well as inflammation and pain. The target scope of Honghua was broader than that of Danggui. Several targets mediated by the active ingredients of Honghua including PTAFR, PTGS2, NOS3 and STAT1, may alleviate the accompanying symptoms of BSS such as inflammation and pain. As to Danggui, its active ingredients mediated several targets including ADRB1, CHRM2, GSK3B, BCL2 and ICAM1, which may be helpful for improving the immune system. Modern research has demonstrated that ischemic processes release mediators activating the innate immune system that may be induced by the formation of thrombi inside blood vessels^53^. Fortuitously, several targets associated with cancer were also discerned in the T-D network. HSYA from Honghua was validated to antagonize tumor angiogenesis by inhibiting the protein expression of VEGFA, MMP9 and HIF1A^4^. Additionally, *Z-*butylidenephthalide, senkyunolide A and *Z*-ligustilide from Danggui were confirmed to exhibit anti-proliferative potential and significant synergy on colon cancer cells^54^.

**Figure 5:**
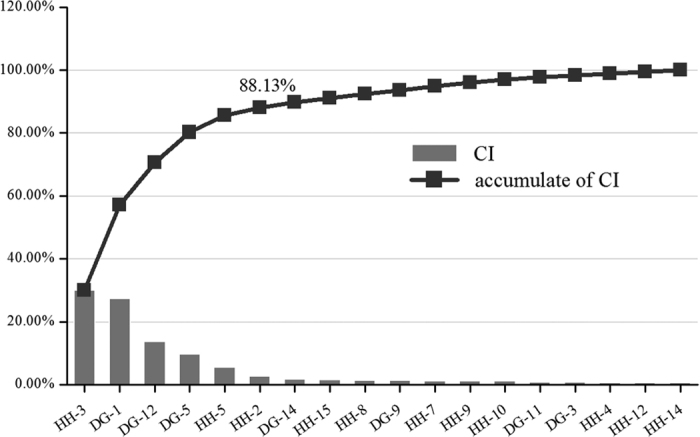
The CI and accumulative CI of active ingredients in Danggui-Honghua.

As mentioned earlier, a contribution index of every active ingredient was proposed based on network based efficacy weighted by literature. According to calculated results (Fig. 5 and Supplementary Table S3), six compounds emerged from the active ingredients, including HSYA, safflor yellow A, safflor yellow B, *Z*-ligustilide, ferulic acid, and *Z-*butylidenephthalide. They displayed the most contribution to the blood-activating and stasis-dissolving effects of Danggui-Honghua with a sum of CIs of 88.13%. Therefore, the above discussion may fully clarify why Danggui-Honghua could produce synergistic and complementary effects.

### Pathway analysis to explore the underlying mechanisms of Danggui-Honghua

**Figure 6:**
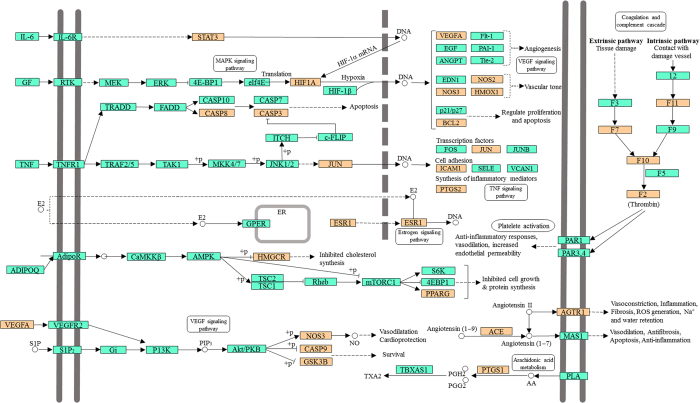
Distribution of partial targets of Danggui-Honghua on the compressed pathway. The orange nodes are potential targets. And the light blue nodes are relevant targets in the pathway.

Signaling pathways, as an important component of the system pharmacology, link receptor-ligand interactions to pharmacodynamics outputs^55^. The canonical pathways associated with BSS treatment and prophylaxis were extracted from Kyoto Encyclopedia of Genes and Genomes (KEGG, http://www.genome.jp/kegg/) database, which ends up with 20 KEGG pathways, including cAMP signaling pathway, calcium signaling pathway, NF-κB signaling pathway and sphingolipid signaling pathway (Fig. 6). The NF-κB signaling pathway is crucial for focal cerebral ischemia/reperfusion induced inflammatory injury^56^. Previous research has shown that HSYA, safflor yellow A, and caffeic acid could inhibit the NF-κB signaling pathway contributing to the cross-talk of multiple targets in anti-inflammation^57^. The sphingolipid signaling pathway was proven to play a critical role in the ischemic preconditioning and the pathophysiology of stroke^58^.

**Figure 7:**
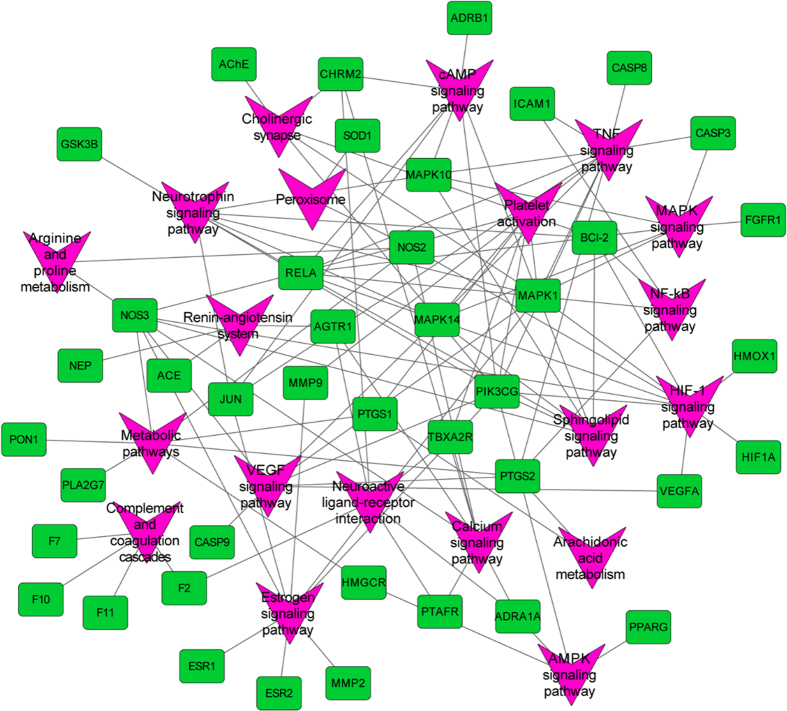
Target-pathway network of Danggui-Honghua where light green nodes represent the targets and purple nodes the pathways.

For the purpose of systematically dissecting the underlying mechanisms of Danggui-Honghua, all of the targets interacting with the active ingredients were mapped onto the 20 KEGG pathways and the T-P network was generated (Fig. 7). The TNF signaling pathway exhibited the highest number of target connections (degree = 11), followed by HIF-1 signaling pathway with 9 targets, estrogen signaling pathway and neurotrophin signaling pathway with 8 ones, respectively. These high-degree pathways were closely related to the vascular and central neural systems and inflammation. The TNF signaling pathway plays an important role in the ischemic stroke and the vascular injury involved in multiple targets including JUN, PTGS2 and ICAM1^59^,^60^. The HIF-1 signaling pathway was an underlying mechanism of neuroprotection and anti-ventricular cell apoptosis^61^,^62^. Fortunately, 22 out of 31 active compounds from Danggui-Honghua, especially HSYA, quercetin, *Z*-ligustilide and luteolin, were implicated in regulating the major targets of HIF-1 signaling pathway, such as HMOX1, NOS2, NOS3, RELA, PIK3CG and MAPK1. Recently, we successfully discovered the underlying blood-activating mechanisms of Danggui-Honghua by metabolomics analysis. Four potential metabolic pathways were speculated, including phenylalanine metabolism, sphingolipid metabolism, arachidonic acid metabolism, and arginine and proline metabolism^63^. In the present study, arginine and proline metabolism, arachidonic acid metabolism, and sphingolipid signaling pathway (including sphingolipid metabolism) were also deciphered, which were consistent with our previous metabolomics results^63^.

Overall, it could be speculated that the herb pair Danggui-Honghua exert the blood-activating and stasis-dissolving effects mainly through the regulation of TNF, HIF-1, estrogen, and neurotrophin signaling pathways. As a holistic medicine, this herb pair may also be implicated in arginine and proline metabolism, arachidonic acid metabolism, VEGF signaling pathway, MAPK signaling pathway, calcium signaling pathway, and sphingolipid signaling pathway to regulate the vascular and nervous systems, as well as the inflammation and pain.

## Discussion

Herb pairs, the smallest compatible units in TCM formulae, have become a prominent concern during the past decade^2^. Danggui and Honghua, combined as a classical herb pair, have been frequently used in TCM prescriptions^5^. Through data mining and pharmacological approaches, this herb pair was found to produce synergistic and complementary effects to treat BSS^64^,^65^,^66^,^67^,^68^,^69^. However, the complexity of the chemical components of this herb pair and their corresponding multiple targets *in vivo* led to extreme difficulty to elucidate the molecular mechanisms.

Nowadays, system pharmacology provides a powerful avenue for compatible and mechanistic exploration of TCM^10^,^11^. In our work, an integrated system pharmacology approach, combined a number of network-based computational methods and algorithm-based approaches, was used to select active compounds, predict targets, construct networks, and illuminate the molecular synergy of Danggui-Honghua on BSS. Thirty-one active ingredients with favorable bioactivities and contents were selected from the 347 compounds of Danggui-Honghua by ADME filtering, providing foundational clues for thorough investigation on this herb pair. It was found that some biological activities against BSS of these active ingredients have been reported previously^26^,^27^,^28^,^39^,^40^,^41^,^42^,^70^,^71^,^72^, highlighting the credibility of our ADME filtering criteria. Then, an integrated *in silico* approach was applied to decipher the 42 targets for these active ingredients related to BSS including 27 common targets, which distinctly explained the action modes and biological processes that active ingredients achieve their synergistic and complementary curative effects. It is worth mentioning that the results from several compound-target interaction experiments by us and other groups indicated the reasonability of our integrated *in silico* approach^45^,^46^,^47^,^48^,^49^,^50^,^51^,^73^. Subsequently, 20 signaling pathways associated with BSS treatment and prophylaxis by Danggui-Honghua were retrieved. A recent study on the anti-inflammatory effects of volatile oils from Danggui (78.61% *Z*-Ligustilide and 7.99% *Z*-Butylidenephthalide) using GC-MS-based metabolomics revealed that it may be involved in regulating the arachidonic acid metabolism^74^, which is comparable to our findings. Finally, the C-T, T-D and T-P networks clearly elucidated the molecular synergistic actions of Danggui-Honghua in a holistic context. By network systematic analysis and contribution index calculation, HSYA, safflor yellow A and safflor yellow B in Honghua, together with *Z*-ligustilide, ferulic acid and *Z-*butylidenephthalide in Danggui, displayed the most contribution to the blood-activating and stasis-dissolving effects of Danggui-Honghua. Meanwhile, this herb pair could regulate the proteins related to vascular and central neural system diseases as well as inflammation and pain implicated in TNF, HIF-1, estrogen, and neurotrophin signaling pathways, arginine and proline metabolism, arachidonic acid metabolism, and VEGF signaling pathway. Noteworthy, based on our research, several targets and signaling pathways of Danggui-Honghua acting on BSS have been found for the first time. All of these results are expected to help identify novel curative efficacy and take full clinical advantage of Danggui-Honghua.

Due to the above findings mainly relied on theoretical analyses, more experiments are anticipated to support these findings as well as potential clinical applications. It should be noted that the majority of the ingredients in both Danggui and Honghua were hydrotropic, and the OB values of a third of the selected 31 active ingredients were less than 30%. Therefore, the availability of these active constituents by gut microbiota especially under the disease state may be a critical step towards the emergence of their bioactivities *in vivo*^75^.

## Methods

### Chemical ingredients database building

All of the constituent data of Danggui (the radix of *A. sinensis*) and Honghua (the florets of *C. tinctorius*) were retrieved from TcmSP™, a unique system pharmacology platform designed for herbal medicines^12^, and then manually supplemented through a wide-scale text-mining method. Meanwhile, four important pharmacology-related properties were also obtained from TcmSP™, including MW, MLogP, nHDon and nHAcc.

### Active ingredients screening

The active ingredients from Danggui-Honghua were filtered by integrating three indexes including OB, Caco-2, and DL. A robust *in silico* model OBioavail 1.1 (Yangling, Shaanxi, China) that integrated the metabolism and transport information was employed to calculate the OB values of all herbal ingredients^76^. Those ingredients with OB ≥ 30% were selected. The VolSurf built-in Caco-2 permeability model (Tripos, St. Louis, America)^77^ was implemented to screen active compounds. Given that molecule with Caco-2 value less than −0.4 is not permeable, the threshold of Caco-2 permeability was set to −0.4. Database-dependent DL evaluation approach based on Tanimoto coefficient^78^ was applied and shown as follows.


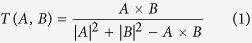


In this eq. (1), A represents the molecular descriptors of herbal compounds, and B displays the average molecular properties of all compounds in Drugbank. Those ingredients with DL ≥ 0.18 were preserved. The ingredients were adopted as the candidate compounds for further analysis when they met all of these three criteria.

### Targets fishing

To identify the corresponding targets of the active ingredients of Danggui-Honghua, several approaches combined with chemometric method, information integration and data-mining were implemented. First of all, the biological targets of active ingredients were obtained from SEA^29^, STITCH^30^, and PharmMapper sever^31^. Amongst them, the PharmMapper gives the best mapping poses by comparing with available targets in PharmTargetDB (a large, in-house repertoire of pharmacophore database belongs to PharmMapper) and the respective N-best fit poses are generated. All active compounds were also sent to HIT^32^, TTD^33^, DrugBank and Google Scholar to mine compound-target interactions supported by literature. Then, to better dissect the role of Danggui-Honghua in BSS treatment, all targets obtained from the previous two steps were sent to TTD, Comparative Toxicogenomics Database (CTD, http://ctdbase.org/) and PharmGKB (http://www.pharmgkb.org)^79^ to mine target-related diseases. Finally, those targets which were implicated in the aforementioned pathophysiology and clinical manifestations of BSS were retained, and the others were eliminated.

### Networks construction

Three networks were constructed: (1) Compound-target network (C-T network). Active ingredients of Danggui-Honghua and their corresponding targets were employed to generate the C-T network. (2) Target-disease network (T-D network). All targets and their corresponding diseases were employed to build a bipartite graph of T-D network. (3) Target-pathway network (T-P network). The pathway information of targets were extracted from the database of KEGG, and then a bipartite T-P network composed of targets and their corresponding putative pathways was built. All visualized network graphs were constructed by Cytoscape 3.2.1 (http://www.cytoscape.org/), an open software package project for visualizing, integrating, modeling and analyzing the interaction networks^80^.

### Contribution indexes calculation

In order to estimate the contribution of each active ingredient to the blood-activating and stasis-dissolving effects of Danggui-Honghua, a contribution index (CI) based on network based efficacy (NE) weighted by literature was proposed and calculated by eqs (2) and (3):


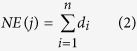



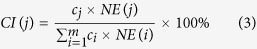


where *n* is the number of targets associated with ingredient *j*; *d*_*i*_ is the degree of target *i* associated with ingredient *j*; *c*_*i*_ is the number of BSS-related literature of ingredient *i*; *m* is the number of ingredients.

If the sum of CIs for the top *N* ingredients was more than 85%, these relevant *N* ingredients were considered to contribute the most to the blood-activating and stasis-dissolving effects.

## Additional Information

**How to cite this article**: Yue, S.-J. *et al*. Herb pair Danggui-Honghua: mechanisms underlying blood stasis syndrome by system pharmacology approach. *Sci. Rep.* **7**, 40318; doi: 10.1038/srep40318 (2017).

**Publisher's note:** Springer Nature remains neutral with regard to jurisdictional claims in published maps and institutional affiliations.

## Supplementary Material

Supplementary Information

## Figures and Tables

**Table 1:** Active ingredients and ADME parameters of Danggui-Honghua.

No.	Name	Structure	OB (%)	Caco-2	DL	Herbs
DG-1	*Z*-Ligustilide	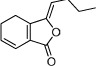	51.30	1.31	0.07	*A. sinensis*
DG-2	Senkyunolide A	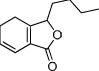	68.28	1.30	0.07	*A. sinensis*
DG-3	Senkyunolide I	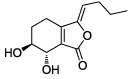	46.80	1.00	0.08	*A. sinensis*
DG-4	Senkyunolide K	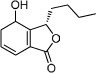	61.75	0.52	0.08	*A. sinensis*
DG-5	*Z*-Butylidenephthalide	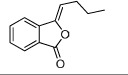	42.44	1.32	0.07	*A. sinensis*
DG-6	3-Butylidene-7-hydroxyphthalide	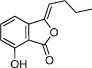	62.68	1.00	0.08	*A. sinensis*
DG-7	Neocnidilide	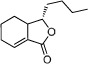	83.83	1.23	0.07	*A. sinensis*
DG-8	Nicotinic acid	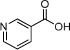	47.65	0.34	0.02	*A. sinensis*
DG-9	Coniferyl ferulate	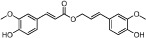	4.54	0.71	0.39	*A. sinensis*
DG-10	Folic acid	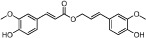	68.96	−1.50	0.71	*A. sinensis*
DG-11	Nodakenin	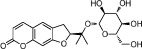	57.12	−0.79	0.69	*A. sinensis*
DG-12	Ferulic acid	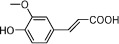	39.56	0.47	0.06	*A. sinensis*
DG-13	Vanillin	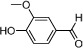	55.14	0.41	0.06	*A. sinensis*
DG-14	Caffeic acid	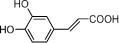	54.97	0.21	0.19	*A. sinensis*
DG-15	*β*-Sitosterol	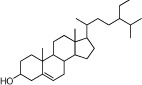	36.91	0.33	0.75	*A. sinensis*
HH-1	Carthamone	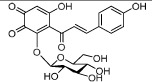	5.93	−1.81	0.63	*C. tinctorius*
HH-2	Safflor yellow A	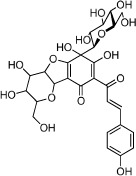	22.75	−2.52	0.75	*C. tinctorius*
HH-3	Hydroxysafflor yellow A	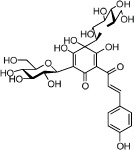	4.77	−2.77	0.68	*C. tinctorius*
HH-4	Precarthamin	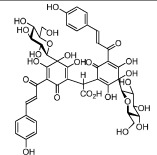	22.00	−1.40	0.67	*C. tinctorius*
HH-5	Safflor yellow B	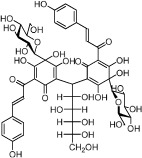	12.00	−2.40	0.67	*C. tinctorius*
HH-6	6-Hydroxykaempferol	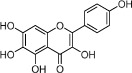	62.13	0.16	0.27	*C. tinctorius*
HH-7	Kaempferol	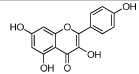	67.43	0.26	0.24	*C. tinctorius*
HH-8	Nicotiflorin	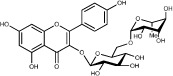	3.64	−1.77	0.73	*C. tinctorius*
HH-9	Quercetin	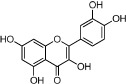	46.43	0.05	0.28	*C. tinctorius*
HH-10	Rutin	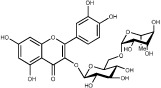	11.70	−1.93	0.68	*C. tinctorius*
HH-11	Eriodictyol	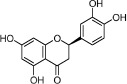	71.79	0.17	0.24	*C. tinctorius*
HH-12	Scutellarin	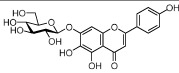	2.64	−1.08	0.79	*C. tinctorius*
HH-13	Scutellarein	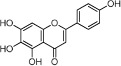	18.97	0.31	0.24	*C. tinctorius*
HH-14	Acacetin	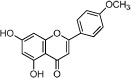	34.97	0.67	0.24	*C. tinctorius*
HH-15	Luteolin	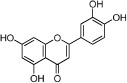	36.16	0.19	0.25	*C. tinctorius*
HH-16	Rosmarinic acid	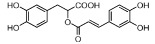	48.60	0.82	0.46	*C. tinctorius*

Note: DG-: the active compound from Danggui (the radix of *A. sinensis*), HH-: the active compound from Honghua (the florets of *C. tinctorius*).

**Table 2:** Target information of Danggui-Honghua.

ID	Target	UniProt ID	Gene name	Related diseases
T-01	Estrogen receptor	P03372	ESR1	Nervous system diseases, Cardiovascular disease, Brain injury, Hyperlipidemia
T-02	Estrogen receptor beta	Q92731	ESR2	Cardiovascular disease, Vascular injury response, Neoplasms
T-03	Peroxisome proliferator activated receptor gamma	P37231	PPARG	Inflammation, Ischemic heart disease
T-04	Superoxide dismutase [Cu-Zn]	P00441	SOD1	Ischemic injury, Neoplasms
T-05	Prostaglandin G/H synthase 1	P23219	PTGS1	Cardiovascular disease, Chronic inflammatory diseases
T-06	Prostaglandin G/H synthase 2	P35354	PTGS2	Inflammation, Nervous system diseases, Myocardial infarction, Stroke, Pain
T-07	Transcription factor AP-1	P05412	JUN	Vascular disease, Immune system diseases, Cancer
T-08	Intercellular adhesion molecule 1	P05362	ICAM1	Inflammation, Cardiovascular disease, Asthma, Autoimmune diseases
T-09	Signal transducer and activator of transcription 1-alpha/beta	P42224	STAT1	Ischemic injury, Inflammatory disorders, Myocardial ischemia and reperfusion injury
T-10	Hypoxia-inducible factor 1-alpha	Q16665	HIF1A	Stroke, Cardiovascular diseases
T-11	Vascular endothelial growth factor A	P15692	VEGFA	Neoplasms, Ischemic heart disease, Coronary artery disease
T-12	Nitric oxide synthase, inducible	P35228	NOS2	Ischemia reperfusion injuries
T-13	Nitric oxide synthase, endothelial	P29474	NOS3	Cardiovascular disease, Inflammation, Coronary artery disease, Angina
T-14	Prothrombin	P00734	F2	Coagulative disorders, Thromboembolic disorders, Coronary atherosclerosis, Thrombosis, Myocardial infarction
T-15	Coagulation factor VII	P08709	F7	Coagulative disorders, Cardiovascular disease, Thromboembolism
T-16	Coagulation factor Xa	P00742	F10	Cardiovascular disease, Coagulative disorders
T-17	Coagulation factor XI	P03951	F11	Clotting Disorders
T-18	Thromboxane A2 receptor	P21731	TBXA2R	Platelet adhesion
T-19	Heme oxygenase 1	P09601	HMOX1	Cardiovascular disease, Ischemic injury of the liver, Inflammation, Vascular disease, Cerebral vasospasm
T-20	Phosphatidylinositol-4,5-bisphosphate 3-kinase catalytic subunit, gamma isoform	P48736	PIK3CG	Heart failure, Myocardial infarction, Cancer, Angioedema
T-21	Platelet-activating factor acetylhydrolase	Q13093	PLA2G7	Atherosclerosis, Cardiovascular disorders
T-22	Type-1 angiotensin II receptor	P30556	AGTR1	Cardiovascular disease, Heart failure, Ischemic stroke, Hypertension
T-23	Apoptosis regulator Bcl-2	P10415	BCL2	Immune system diseases, Cardiovascular diseases
T-24	Transcription factor p65	Q04206	RELA	Embolic focal cerebral ischemia, Ischemic renal injury, Thrombosis, Inflammation, Atherosclerosis
T-25	Matrix metalloproteinase-2	P08253	MMP2	Atherosclerosis, Cancer, Multiple sclerosis, Coronary artery disease
T-26	Matrix metalloproteinase-9	P14780	MMP9	Atherosclerosis, Cancer, Multiple sclerosis, Coronary artery disease, Heart failure
T-27	Acetylcholinesterase	P22303	AChE	Brain ischemia, Nervous system diseases, Cognitive deficits
T-28	Caspase-3	P42574	CASP3	Venous thrombosis
T-29	Caspase-8	Q14790	CASP8	Nervous system diseases
T-30	Caspase-9	P55211	CASP9	Nervous system diseases, Brain injury
T-31	Alpha-1A adrenergic receptor	P35348	ADRA1A	Urogenital system, Hypertrophic vascular disease
T-32	Beta-1 adrenergic receptor	P08588	ADRB1	Cardiac arrhythmias, Cardiovascular disease, Coronary heart disease, Immune system disorders
T-33	Muscarinic acetylcholine receptor M2	P08172	CHRM2	Autoimmune cardiomyopathy, Chronic obstructive pulmonary disease, Pain
T-34	Neprilysin	P08473	NEP	Congestive Heart Failure, Hypertension, Prostate cancer
T-35	Glycogen synthase kinase-3 beta	P49841	GSK3B	Brain injury, Immunodeficiency, Ischemia, Alzheimer’s disease
T-36	3-Hydroxy-3-methylglutaryl-coenzyme A reductase	P13702	HMGCR	Atherosclerosis, Cardiovascular disease, Coronary heart disease, Myocardial infarction
T-37	Mitogen-activated protein kinase 1	P28482	MAPK1	Neurodegenerative diseases, Proliferative diseases
T-38	Mitogen-activated protein kinase 10	P53779	MAPK10	Ischemic stroke, Neurological diseases
T-39	Mitogen-activated protein kinase 14	Q16539	MAPK14	Thrombosis, Inflammation, Alzheimer’s disease
T-40	Angiotensin-converting enzyme	P12821	ACE	Heart failure, Hypertension, Vascular disease
T-41	Platelet activating factor receptor	P25105	PTAFR	Ocular allergy, Hypertension, Inflammation
T-42	Fibroblast growth factor receptor 1	P11362	FGFR1	Peripheral vascular disease, Coronary heart disease, Cancer
