# Supplementary material for: Herb pair Danggui-Honghua: mechanisms underlying blood stasis syndrome by system pharmacology approach
Source: Sci Rep. 2017 Jan 11;7:40318. doi: 10.1038/srep40318 (PMC5225497; doi:10.1038/srep40318)
Supplement: Supplementary Information [file srep40318-s1.doc]

**Herb pair Danggui-Honghua: mechanisms underlying blood stasis syndrome by system pharmacology approach**

**Shi-Jun Yue**^1,2^ ***, Lan-Ting Xin**^1,2^ ***, Ya-Chu Fan**^1,2^**, Shu-Jiao Li**^3^**, Yu-Ping Tang**^3^**, Jin-Ao Duan**^3^**, Hua-Shi Guan**^1,2^**, Chang-Yun Wang**^1,2^

^1^Key Laboratory of Marine Drugs, The Ministry of Education of China, School of Medicine and Pharmacy, Ocean University of China, Qingdao 266003, P. R. China, ^2^Laboratory for Marine Drugs and Bioproducts, Qingdao National Laboratory for Marine Science and Technology, Qingdao 266071, P. R. China, ^3^Jiangsu Key Laboratory for High Technology Research of TCM Formulae, Nanjing University of Chinese Medicine, Nanjing 210023, P. R. China.

Correspondence and requests for materials should be addressed to C.Y.W. (changyun@ouc.edu.cn) or H.S.G. (hsguan@ouc.edu.cn)

* These authors contributed equally to this work.

**Table S1**. The detailed information of all compounds in Danggui-Honghua.

| **ID** | **Herb** | **Compound** | **CAS** | **OB** | **Caco-2** | **DL** | **MW** | **MLogP** | **nHDon** | **nHAcc** |
| --- | --- | --- | --- | --- | --- | --- | --- | --- | --- | --- |
| **0** | *Angelica sinensis* | Butanal | 123-72-8 | 68.66 | 1.18 | 0 | 72.12 | 0.94 | 0 | 1 |
| **1** | *Angelica sinensis* | Methylbutenol | 115-18-4 | 54.58 | 1.12 | 0.01 | 86.15 | 0.84 | 1 | 1 |
| **2** | *Angelica sinensis* | Butyric acid | 107-92-6 | 21.62 | 0.69 | 0.00 | 88.12 | 0.89 | 1 | 2 |
| **3** | *Angelica sinensis* | Hexanal | 66-25-1 | 55.71 | 1.25 | 0.01 | 100.18 | 1.85 | 0 | 1 |
| **4** | *Angelica sinensis* | Tropone | 539-80-0 | 47.41 | 1.25 | 0.01 | 106.13 | 1.14 | 0 | 1 |
| **5** | *Angelica sinensis* | 4-Methylphenol | 106-44-5 | 51.99 | 1.56 | 0.01 | 108.15 | 2.05 | 1 | 1 |
| **6** | *Angelica sinensis* | 2-Methylphenol | 95-48-7 | 62.45 | 1.57 | 0.02 | 108.15 | 2.05 | 1 | 1 |
| **7** | *Angelica sinensis* | Heptanal | 111-71-7 | 19.59 | 1.29 | 0.01 | 114.21 | 2.31 | 0 | 1 |
| **8** | *Angelica sinensis* | Succinic acid | 110-15-6 | 29.62 | -0.44 | 0.01 | 118.10 | -0.41 | 2 | 4 |
| **9** | *Angelica sinensis* | Acetylbenzene | 98-86-2 | 48.19 | 1.36 | 0.02 | 120.16 | 1.57 | 0 | 1 |
| **10** | *Angelica sinensis* | 4-Ethylphenol | 123-07-9 | 48.44 | 1.57 | 0.02 | 122.18 | 2.51 | 1 | 1 |
| **11** | *Angelica sinensis* | *o*-Xylenol | 526-75-0 | 53.13 | 1.62 | 0.02 | 122.18 | 2.54 | 1 | 1 |
| **12** | *Angelica sinensis* | *m*-Ethylphenol | 620-17-7 | 51.30 | 1.55 | 0.02 | 122.18 | 2.51 | 1 | 1 |
| **13** | *Angelica sinensis* | Nicotinic acid | 59-67-6 | 47.65 | 0.34 | 0.02 | 123.12 | 0.28 | 1 | 3 |
| **14** | *Angelica sinensis* | Guaiacol | 90-05-1 | 51.60 | 1.28 | 0.02 | 124.15 | 1.55 | 1 | 2 |
| **15** | *Angelica sinensis* | 4-Methyl-6-hepten-3-one | 26118-97-8 | 78.38 | 1.39 | 0.01 | 126.22 | 2.07 | 0 | 1 |
| **16** | *Angelica sinensis* | 4-Octanone | 589-63-9 | 19.37 | 1.37 | 0.01 | 128.24 | 2.46 | 0 | 1 |
| **17** | *Angelica sinensis* | 5-Hydroxyindole | 1953-54-4 | 63.14 | 1.38 | 0.03 | 133.16 | 1.86 | 2 | 1 |
| **18** | *Angelica sinensis* | 3,4-Dimethylbenzaldehyde | 5973-71-7 | 39.99 | 1.40 | 0.02 | 134.19 | 2.56 | 0 | 1 |
| **19** | *Angelica sinensis* | 4-Ethylbenzaldehyde | 4748-78-1 | 40.95 | 1.40 | 0.02 | 134.19 | 2.53 | 0 | 1 |
| **20** | *Angelica sinensis* | Isoxylaldehyde | 5779-94-2 | 38.85 | 1.39 | 0.02 | 134.19 | 2.56 | 0 | 1 |
| **21** | *Angelica sinensis* | 2,4-Dimethylbenzaldehyde | 15764-16-6 | 39.33 | 1.42 | 0.03 | 134.19 | 2.56 | 0 | 1 |
| **22** | *Angelica sinensis* | 4-Isopropyltoluene | 99-87-6 | 27.20 | 1.86 | 0.02 | 134.24 | 3.51 | 0 | 0 |
| **23** | *Angelica sinensis* | Adenine | 73-24-5 | 62.81 | -0.30 | 0.03 | 135.15 | -0.58 | 3 | 4 |
| **24** | *Angelica sinensis* | (-)-alpha-Pinene | 7785-26-4 | 46.25 | 1.85 | 0.05 | 136.26 | 2.87 | 0 | 0 |
| **25** | *Angelica sinensis* | Myrcene | 123-35-3 | 24.96 | 1.84 | 0.02 | 136.26 | 3.69 | 0 | 0 |
| **26** | *Angelica sinensis* | Ocimene | 3779-61-1 | 15.06 | 1.85 | 0.02 | 136.26 | 3.63 | 0 | 0 |
| **27** | *Angelica sinensis* | Moslene | 99-85-4 | 33.02 | 1.88 | 0.02 | 136.26 | 3.45 | 0 | 0 |
| **28** | *Angelica sinensis* | Allocymene | 673-84-7 | 14.89 | 1.85 | 0.02 | 136.26 | 3.58 | 0 | 0 |
| **29** | *Angelica sinensis* | Citrene | 5989-27-5 | 39.84 | 1.83 | 0.02 | 136.26 | 3.50 | 0 | 0 |
| **30** | *Angelica sinensis* | Isodiprene | 498-15-7 | 45.20 | 1.84 | 0.04 | 136.26 | 2.87 | 0 | 0 |
| **31** | *Angelica sinensis* | *β*-Terpinen | 99-84-3 | 42.29 | 1.85 | 0.02 | 136.26 | 3.50 | 0 | 0 |
| **32** | *Angelica sinensis* | 1,5,5,6-Tetramethyl-1,3-cyclohexadiene | 514-94-3 | 39.22 | 1.82 | 0.03 | 136.26 | 3.00 | 0 | 0 |
| **33** | *Angelica sinensis* | 4-Hydroxybenzoic acid | 99-96-7 | 30.15 | 0.39 | 0.03 | 138.13 | 1.17 | 2 | 3 |
| **34** | *Angelica sinensis* | 6-Ethylresorcinol | 2896-60-8 | 46.45 | 1.13 | 0.03 | 138.18 | 2.24 | 2 | 2 |
| **35** | *Angelica sinensis* | Nonanal | 124-19-6 | 40.28 | 1.31 | 0.02 | 142.27 | 3.22 | 0 | 1 |
| **36** | *Angelica sinensis* | 1,3-Phthalandione | 85-44-9 | 47.31 | 0.56 | 0.04 | 148.12 | 1.30 | 0 | 3 |
| **37** | *Angelica sinensis* | Mesitaldehyde | 487-68-3 | 37.80 | 1.54 | 0.03 | 148.22 | 3.05 | 0 | 1 |
| **38** | *Angelica sinensis* | 4-Isopropylbenzaldehyde | 122-03-2 | 38.29 | 1.39 | 0.03 | 148.22 | 2.78 | 0 | 1 |
| **39** | *Angelica sinensis* | Isoamylbenzene | 2049-94-7 | 35.69 | 1.84 | 0.03 | 148.27 | 3.94 | 0 | 0 |
| **40** | *Angelica sinensis* | *L*-Met | 3654-96-4 | 70.87 | 0.06 | 0.01 | 149.24 | -0.27 | 3 | 3 |
| **41** | *Angelica sinensis* | 2-Acetyl-4-methylphenol | 1450-72-2 | 24.96 | 1.02 | 0.03 | 150.19 | 1.79 | 1 | 2 |
| **42** | *Angelica sinensis* | Verbenone | 80-57-9 | 50.63 | 1.27 | 0.06 | 150.24 | 1.94 | 0 | 1 |
| **43** | *Angelica sinensis* | Carvacrol | 499-75-2 | 43.28 | 1.58 | 0.03 | 150.24 | 3.24 | 1 | 1 |
| **44** | *Angelica sinensis* | Eucarvone | 503-93-5 | 53.14 | 1.35 | 0.03 | 150.24 | 2.07 | 0 | 1 |
| **45** | *Angelica sinensis* | (*R*)-6-Butyl-1,4-cycloheptadiene | 33156-91-1 | 31.69 | 1.85 | 0.02 | 150.29 | 3.93 | 0 | 0 |
| **46** | *Angelica sinensis* | 4-Methoxybenzoic acid | 100-09-4 | 29.69 | 0.69 | 0.03 | 152.16 | 1.42 | 1 | 3 |
| **47** | *Angelica sinensis* | vanillin | 121-33-5 | 55.14 | 0.68 | 0.03 | 152.16 | 1.31 | 1 | 3 |
| **48** | *Angelica sinensis* | Resacetophenone | 89-84-9 | 36.49 | 0.67 | 0.03 | 152.16 | 1.03 | 2 | 3 |
| **49** | *Angelica sinensis* | 2,3,5-Trimethylbenzene-1,4-diol | 700-13-0 | 54.42 | 1.21 | 0.03 | 152.21 | 2.75 | 2 | 2 |
| **50** | *Angelica sinensis* | Protocatechuic acid | 99-50-3 | 25.37 | 0.10 | 0.04 | 154.13 | 0.90 | 3 | 4 |
| **51** | *Angelica sinensis* | (*R*)-Alpha-terpineol | 98-55-5 | 46.30 | 1.28 | 0.03 | 154.28 | 2.42 | 1 | 1 |
| **52** | *Angelica sinensis* | Decanal | 112-31-2 | 29.81 | 1.34 | 0.02 | 156.30 | 3.68 | 0 | 1 |
| **53** | *Angelica sinensis* | Undecane | 1120-21-4 | 17.15 | 1.79 | 0.02 | 156.35 | 5.39 | 0 | 0 |
| **54** | *Angelica sinensis* | 2,4,6-Trimethyl-octane | 62016-37-9 | 29.14 | 1.81 | 0.02 | 156.35 | 4.78 | 0 | 0 |
| **55** | *Angelica sinensis* | 3,7-Dimethyl-nonane | 17302-32-8 | 16.97 | 1.78 | 0.02 | 156.35 | 4.98 | 0 | 0 |
| **56** | *Angelica sinensis* | Safrole | 94-59-7 | 45.34 | 1.44 | 0.05 | 162.20 | 2.61 | 0 | 2 |
| **57** | *Angelica sinensis* | Isoeugenol | 97-54-1 | 70.10 | 1.38 | 0.04 | 164.22 | 2.50 | 1 | 2 |
| **58** | *Angelica sinensis* | *cis*-Isoeugenol | 5912-86-7 | 20.73 | 1.43 | 0.04 | 164.22 | 2.50 | 1 | 2 |
| **59** | *Angelica sinensis* | 1,1,5-Trimethyl-2-formylcyclohexa-2,5-diene-4-one | 23985-22-0 | 48.94 | 0.82 | 0.04 | 164.22 | 1.64 | 0 | 2 |
| **60** | *Angelica sinensis* | Phthalic acid | 88-99-3 | 17.74 | -0.05 | 0.04 | 166.14 | 1.04 | 2 | 4 |
| **61** | *Angelica sinensis* | Vanillic acid | 121-34-6 | 35.47 | 0.43 | 0.04 | 168.16 | 1.15 | 2 | 4 |
| **62** | *Angelica sinensis* | Dodecene | 112-41-4 | 17.74 | 1.80 | 0.02 | 168.36 | 5.46 | 0 | 0 |
| **63** | *Angelica sinensis* | 6-Undecanone | 927-49-1 | 18.65 | 1.31 | 0.02 | 170.33 | 3.83 | 0 | 1 |
| **64** | *Angelica sinensis* | Dodecane | 112-40-3 | 17.74 | 1.79 | 0.02 | 170.38 | 5.85 | 0 | 0 |
| **65** | *Angelica sinensis* | Decanoic acid | 334-48-5 | 26.74 | 0.96 | 0.03 | 172.30 | 3.63 | 1 | 2 |
| **66** | *Angelica sinensis* | Undecanol-6 | 23708-56-7 | 25.77 | 0.96 | 0.02 | 172.35 | 4.15 | 1 | 1 |
| **67** | *Angelica sinensis* | Caffeic acid | 331-39-5 | 54.97 | 0.27 | 0.05 | 180.17 | 1.37 | 3 | 4 |
| **68** | *Angelica sinensis* | Tridecene | 2437-56-1 | 17.69 | 1.83 | 0.03 | 182.39 | 5.91 | 0 | 0 |
| **69** | *Angelica sinensis* | *Z-*Butylidenephthalide | 551-08-6 | 42.44 | 1.32 | 0.07 | 188.24 | 3.00 | 0 | 2 |
| **70** | *Angelica sinensis* | *Z*-Ligustilide | 4431-01-0 | 51.30 | 1.30 | 0.07 | 190.26 | 2.94 | 0 | 2 |
| **71** | *Angelica sinensis* | (3*S*)-butylphthalide | 6066-49-5 | 55.05 | 1.30 | 0.07 | 190.26 | 3.25 | 0 | 2 |
| **72** | *Angelica sinensis* | Scopoletin | 92-61-5 | 27.77 | 0.71 | 0.08 | 192.18 | 1.62 | 1 | 4 |
| **73** | *Angelica sinensis* | Senkyunolide A | 63038-10-8 | 68.28 | 1.28 | 0.07 | 192.28 | 3.19 | 0 | 2 |
| **74** | *Angelica sinensis* | *D*-Galacturonic acid | 11100-11-1 | 29.75 | -2.00 | 0.04 | 194.16 | -2.47 | 5 | 7 |
| **75** | *Angelica sinensis* | Ferulic acid | 537-98-4 | 39.56 | 0.47 | 0.06 | 194.20 | 1.62 | 2 | 4 |
| **76** | *Angelica sinensis* | *E*-Ferulic acid | 1014-83-1 | 54.97 | 0.53 | 0.06 | 194.20 | 1.62 | 2 | 4 |
| **77** | *Angelica sinensis* | Sedanolide | 6415-59-4 | 62.46 | 1.24 | 0.07 | 194.30 | 3.37 | 0 | 2 |
| **78** | *Angelica sinensis* | Neocnidilide | 4567-33-3 | 83.83 | 1.23 | 0.07 | 194.30 | 3.37 | 0 | 2 |
| **79** | *Angelica sinensis* | (*Z*)-2-[[(*Z*)-2-methylbut-2-enoyl]oxymethyl]but-2-enoic acid | 69188-40-5 | 77.10 | 0.49 | 0.04 | 198.24 | 2.15 | 1 | 4 |
| **80** | *Angelica sinensis* | (*Z*)-2-Hexenyl hexanoate | 53398-86-0 | 19.39 | 1.26 | 0.04 | 198.34 | 3.99 | 0 | 2 |
| **81** | *Angelica sinensis* | Camphoric acid | 560-09-8 | 99.13 | 0.10 | 0.07 | 200.26 | 1.48 | 2 | 4 |
| **82** | *Angelica sinensis* | Sebacic acid | 111-20-6 | 16.23 | -0.01 | 0.05 | 202.28 | 2.33 | 2 | 4 |
| **83** | *Angelica sinensis* | (*R*)-Cuparene | 16982-00-6 | 38.26 | 1.88 | 0.07 | 202.37 | 4.72 | 0 | 0 |
| **84** | *Angelica sinensis* | 3-Butylidene-7-hydroxyphthalide | 93236-67-0 | 62.68 | 1.00 | 0.08 | 204.24 | 2.74 | 1 | 3 |
| **85** | *Angelica sinensis* | Senkyunolide I | 63038-10-8 | 46.80 | 0.87 | 0.08 | 204.24 | 2.74 | 1 | 3 |
| **86** | *Angelica sinensis* | Senkyunolide E | 91652-78-7 | 34.40 | 0.55 | 0.08 | 204.24 | 1.90 | 1 | 3 |
| **87** | *Angelica sinensis* | Senkyunolide H | 91652-78-7 | 34.40 | 0.55 | 0.08 | 204.24 | 1.90 | 1 | 3 |
| **88** | *Angelica sinensis* | (3*E*)-3-Butylidene-7-hydroxy-2-benzofuran-1-one | 103659-69-4 | 42.17 | 1.03 | 0.08 | 204.24 | 2.74 | 1 | 3 |
| **89** | *Angelica sinensis* | *β*-Chamigrene | 18431-82-8 | 31.99 | 1.82 | 0.08 | 204.39 | 4.71 | 0 | 0 |
| **90** | *Angelica sinensis* | *E*-Thujopsene | 470-40-6 | 56.43 | 1.84 | 0.12 | 204.39 | 4.08 | 0 | 0 |
| **91** | *Angelica sinensis* | *β*-Bisabolene | 495-61-4 | 20.30 | 1.89 | 0.06 | 204.39 | 5.33 | 0 | 0 |
| **92** | *Angelica sinensis* | *β*-Funebrene | 50894-66-1 | 52.87 | 1.79 | 0.1 | 204.39 | 4.12 | 0 | 0 |
| **93** | *Angelica sinensis* | *β*-Selinene | 17066-67-0 | 24.39 | 1.83 | 0.08 | 204.39 | 4.81 | 0 | 0 |
| **94** | *Angelica sinensis* | *β*-Cadinene | 523-47-7 | 17.12 | 1.88 | 0.08 | 204.39 | 4.75 | 0 | 0 |
| **95** | *Angelica sinensis* | *α*-Acoradiene | 24048-44-0 | 36.73 | 1.85 | 0.07 | 204.39 | 4.75 | 0 | 0 |
| **96** | *Angelica sinensis* | (*E*)-*β*-farnesene | 502-60-3 | 17.42 | 1.95 | 0.05 | 204.39 | 5.52 | 0 | 0 |
| **97** | *Angelica sinensis* | *γ*-Cadinene | 1460-97-5 | 19.80 | 1.86 | 0.08 | 204.39 | 4.80 | 0 | 0 |
| **98** | *Angelica sinensis* | Bicycloelemene | 32531-56-9 | 20.89 | 1.88 | 0.08 | 204.39 | 4.89 | 0 | 0 |
| **99** | *Angelica sinensis* | *α*-acoradiene | 28400-13-7 | 40.98 | 1.82 | 0.07 | 204.39 | 4.51 | 0 | 0 |
| **100** | *Angelica sinensis* | *α*-Cedrene | 469-61-4 | 55.56 | 1.79 | 0.10 | 204.39 | 4.12 | 0 | 0 |
| **101** | *Angelica sinensis* | *α*-Copaene | 3856-25-5 | 29.33 | 1.83 | 0.12 | 204.39 | 4.17 | 0 | 0 |
| **102** | *Angelica sinensis* | 4-epi-*α*-Acoradiene | 959299-80-0 | 40.65 | 1.83 | 0.07 | 204.39 | 4.75 | 0 | 0 |
| **103** | *Angelica sinensis* | Aromadendrene | 489-39-4 | 18.21 | 1.83 | 0.10 | 204.39 | 4.22 | 0 | 0 |
| **104** | *Angelica sinensis* | (+)-endo-*β*-Bergamotene | 15438-93-4 | 28.51 | 1.86 | 0.09 | 204.39 | 4.75 | 0 | 0 |
| **105** | *Angelica sinensis* | Bicyclogermacrene | 24703-35-3 | 30.73 | 1.86 | 0.08 | 204.39 | 4.7 | 0 | 0 |
| **106** | *Angelica sinensis* | Senkyunolide C | 63038-10-8 | 46.80 | 0.87 | 0.08 | 204.39 | 2.74 | 1 | 3 |
| **107** | *Angelica sinensis* | 2-Valerylbenzoic acid | 64624-87-9 | 78.26 | 0.61 | 0.06 | 206.26 | 2.75 | 1 | 3 |
| **108** | *Angelica sinensis* | Senkyunolide F | 94530-84-4 | 40.35 | 0.61 | 0.08 | 206.26 | 1.84 | 1 | 3 |
| **109** | *Angelica sinensis* | Senkyunolide K | 114569-33-4 | 61.75 | 0.52 | 0.08 | 208.28 | 2.09 | 1 | 3 |
| **110** | *Angelica sinensis* | Senkyunolide G | 94530-85-5 | 39.52 | 0.63 | 0.08 | 208.28 | 2.54 | 1 | 3 |
| **111** | *Angelica sinensis* | (1*R*,2*S*,4*R*)-1-Ethyl-1-methyl-2,4-bis(1-methylethyl)cyclohexane | 515-12-8 | 15.01 | 1.80 | 0.06 | 210.45 | 5.57 | 0 | 0 |
| **112** | *Angelica sinensis* | Tetradecanol | 112-72-1 | 14.19 | 1.27 | 0.05 | 214.44 | 5.53 | 1 | 1 |
| **113** | *Angelica sinensis* | Spathulenol | 6750-60-3 | 80.01 | 1.26 | 0.12 | 220.39 | 3.01 | 1 | 1 |
| **114** | *Angelica sinensis* | Senkyunolide D | 93236-67-0 | 79.13 | 0.12 | 0.10 | 222.26 | 1.80 | 1 | 4 |
| **115** | *Angelica sinensis* | (+)-Ledol | 577-27-5 | 16.96 | 1.43 | 0.12 | 222.41 | 3.20 | 1 | 1 |
| **116** | *Angelica sinensis* | Phosphatdic acid | 97281-52-2 | 19.32 | -1.22 | 0.05 | 228.11 | -1.55 | 2 | 8 |
| **117** | *Angelica sinensis* | Phosphatidylinositol_qt | 383907-36-6 | 12.66 | -1.15 | 0.05 | 228.11 | -1.55 | 2 | 8 |
| **118** | *Angelica sinensis* | Pentadecanol | 629-76-5 | 13.73 | 1.30 | 0.06 | 228.47 | 5.99 | 1 | 1 |
| **119** | *Angelica sinensis* | 7,10-Pentadecadiynoic acid | 22117-06-2 | 41.50 | 1.32 | 0.09 | 234.37 | 5.27 | 1 | 2 |
| **120** | *Angelica sinensis* | Hexadecanol | 124-29-8 | 13.32 | 1.31 | 0.08 | 242.50 | 6.45 | 1 | 1 |
| **121** | *Angelica sinensis* | Senkyunolide L | 114586-51-5 | 29.64 | 0.63 | 0.09 | 242.72 | 2.52 | 1 | 3 |
| **122** | *Angelica sinensis* | (*E*)-3-Octadecene | 7206-19-1 | 19.50 | 1.86 | 0.09 | 252.54 | 8.14 | 0 | 0 |
| **123** | *Angelica sinensis* | Palmitic acid | 57-10-3 | 19.30 | 1.09 | 0.10 | 256.48 | 6.37 | 1 | 2 |
| **124** | *Angelica sinensis* | 2-Methylhexadecanoic acid | 2490-53-1 | 20.23 | 1.07 | 0.11 | 270.51 | 6.83 | 1 | 2 |
| **125** | *Angelica sinensis* | 2,6-di-(phenyl)Thiopyran-4-thione | 1029-97-6 | 69.13 | 1.74 | 0.15 | 280.43 | 4.62 | 0 | 0 |
| **126** | *Angelica sinensis* | 2-Methyl-5-decanone | 54410-89-8 | 20.40 | 1.42 | 0.11 | 310.63 | 7.99 | 0 | 1 |
| **127** | *Angelica sinensis* | Coniferyl ferulate | 63644-62-2 | 4.54 | 0.71 | 0.39 | 356.40 | 3.64 | 2 | 6 |
| **128** | *Angelica sinensis* | Levistolid A | 88182-33-6 | 2.15 | 0.94 | 0.82 | 380.52 | 4.97 | 0 | 4 |
| **129** | *Angelica sinensis* | 6,7,3',8'-Diligustilide | 354-45-0 | 9.83 | 0.79 | 0.70 | 380.52 | 5.17 | 0 | 4 |
| **130** | *Angelica sinensis* | Senkyunolide P | 142864-23-1 | 9.38 | 0.92 | 0.81 | 382.54 | 5.22 | 0 | 4 |
| **131** | *Angelica sinensis* | Phosphatidylinositol | 383907-36-6 | 4.63 | -2.34 | 0.29 | 390.27 | -3.85 | 6 | 13 |
| **132** | *Angelica sinensis* | Nodakenin | 495-31-8 | 57.12 | -0.79 | 0.69 | 408.44 | 0.28 | 4 | 9 |
| **133** | *Angelica sinensis* | Stigmasterol | 83-48-7 | 43.83 | 1.44 | 0.76 | 412.77 | 7.64 | 1 | 1 |
| **134** | *Angelica sinensis* | *β*-sitosterol | 83-46-5 | 36.91 | 1.32 | 0.75 | 414.79 | 8.08 | 1 | 1 |
| **135** | *Angelica sinensis* | Folic acid | 59-30-3 | 68.96 | -1.50 | 0.71 | 441.45 | 0.01 | 7 | 13 |
| **136** | *Angelica sinensis* | Folinic acid | 21-31-4 | 23.60 | -1.70 | 0.74 | 473.50 | -0.04 | 8 | 14 |
| **137** | *Angelica sinensis* | Sphingomyelin | 85187-10-6 | 0.31 | -0.46 | 0.51 | 493.73 | 3.18 | 3 | 7 |
| **138** | *Angelica sinensis* | Sitogluside | 474-58-8 | 20.63 | -0.14 | 0.62 | 576.95 | 6.34 | 4 | 6 |
| **139** | *Angelica sinensis* | Isotetandrine | 477-57-6 | 10.42 | 0.95 | 0.10 | 622.82 | 7.22 | 0 | 8 |
| **140** | *Angelica sinensis* | 1,2-Dimyristoyl-sn-glycero-3-phosphoethanolamine | 90989-93-8 | 20.69 | -0.43 | 0.47 | 635.97 | 9.49 | 3 | 9 |
| **141** | *Angelica sinensis* | Lecithin | 8002-43-5 | 0.31 | 0.16 | 0.40 | 678.06 | 8.77 | 0 | 8 |
| **142** | *Angelica sinensis* | 4-chloro-N-[1-methyl-5-[[1-methyl-5-[[1-methyl-5-(2-morpholinoethylcarbamoyl)pyrrol-3-yl]carbamoyl]pyrrol-3-yl]carbamoyl]pyrrol-3-yl]-5-[2-(2-pyridyl)ethylamino]isothiazole-3-carboxamide | 210045-96-8 | 7.18 | -0.21 | 0.31 | 762.38 | 2.93 | 5 | 13 |
| **143** | *Carthamus tinctorius* | Glyoxylic acid | 298-12-4 | 32.25 | -0.19 | 0.00 | 74.04 | -0.43 | 1 | 3 |
| **144** | *Carthamus tinctorius* | (3*R*)-Pent-1-en-3-ol | 616-25-1 | 72.18 | 1.08 | 0.00 | 86.15 | 1.16 | 1 | 1 |
| **145** | *Carthamus tinctorius* | Acetoxyethane | 141-78-6 | 45.02 | 1.07 | 0.00 | 88.12 | 0.37 | 0 | 2 |
| **146** | *Carthamus tinctorius* | Z-2-Hexenal | 505-57-7 | 46.01 | 1.29 | 0.01 | 98.16 | 1.83 | 0 | 1 |
| **147** | *Carthamus tinctorius* | Isovaleric acid | 503-74-2 | 62.17 | 0.82 | 0.01 | 102.15 | 1.15 | 1 | 2 |
| **148** | *Carthamus tinctorius* | (2*R*)-2-Methylbutyric acid | 116-53-0 | 28.86 | 0.76 | 0.01 | 102.15 | 1.36 | 1 | 2 |
| **149** | *Carthamus tinctorius* | (*R*)-2-Hexanol | 626-93-7 | 43.94 | 1.11 | 0.01 | 102.20 | 1.80 | 1 | 1 |
| **150** | *Carthamus tinctorius* | (3*R*)-Hexan-3-ol | 623-37-0 | 80.12 | 1.20 | 0.01 | 102.20 | 1.87 | 1 | 1 |
| **151** | *Carthamus tinctorius* | *o*-Xylene | 95-47-6 | 45.55 | 1.85 | 0.01 | 106.18 | 2.80 | 0 | 0 |
| **152** | *Carthamus tinctorius* | *p*-Xylene | 106-42-3 | 48.74 | 1.83 | 0.01 | 106.18 | 2.80 | 0 | 0 |
| **153** | *Carthamus tinctorius* | Phenylethane | 100-41-4 | 49.38 | 1.83 | 0.01 | 106.18 | 2.77 | 0 | 0 |
| **154** | *Carthamus tinctorius* | Catechol | 120-80-9 | 29.86 | 1.09 | 0.02 | 110.12 | 1.30 | 2 | 2 |
| **155** | *Carthamus tinctorius* | Uracil | 66-22-8 | 42.53 | 0.05 | 0.02 | 112.10 | -1.01 | 2 | 4 |
| **156** | *Carthamus tinctorius* | Hexanoic acid | 142-62-1 | 73.08 | 0.80 | 0.01 | 116.18 | 1.81 | 1 | 2 |
| **157** | *Carthamus tinctorius* | (2*R*)-5-Methylhexan-2-ol | 627-59-8 | 23.60 | 1.11 | 0.01 | 116.23 | 2.06 | 1 | 1 |
| **158** | *Carthamus tinctorius* | Succinic acid | 110-15-6 | 29.62 | -0.44 | 0.01 | 118.10 | -0.41 | 2 | 4 |
| **159** | *Carthamus tinctorius* | Hyacinthin | 122-78-1 | 38.65 | 1.31 | 0.02 | 120.16 | 1.52 | 0 | 1 |
| **160** | *Carthamus tinctorius* | 4-Hydroxybenzaldehyde | 123-08-0 | 29.98 | 0.82 | 0.02 | 122.13 | 1.32 | 1 | 2 |
| **161** | *Carthamus tinctorius* | 5-Hydroxymethyl-2-furaldehyde | 67-47-0 | 45.07 | 0.05 | 0.02 | 126.12 | 0.67 | 1 | 3 |
| **162** | *Carthamus tinctorius* | Thymine | 65-71-4 | 74.20 | 0.24 | 0.02 | 126.13 | -0.56 | 2 | 4 |
| **163** | *Carthamus tinctorius* | Adenine | 73-24-5 | 62.81 | -0.30 | 0.03 | 135.15 | -0.58 | 3 | 4 |
| **164** | *Carthamus tinctorius* | Benzothiazole | 95-16-9 | 70.33 | 1.39 | 0.03 | 135.20 | 1.91 | 0 | 1 |
| **165** | *Carthamus tinctorius* | 4-Hydroxyacetophenone | 99-93-4 | 36.80 | 0.87 | 0.03 | 136.16 | 1.30 | 1 | 2 |
| **166** | *Carthamus tinctorius* | Salicylic acid | 69-72-7 | 32.13 | 0.63 | 0.03 | 138.13 | 1.17 | 2 | 3 |
| **167** | *Carthamus tinctorius* | 4-Hydroxybenzoic acid | 99-96-7 | 30.15 | 0.39 | 0.03 | 138.13 | 1.17 | 2 | 3 |
| **168** | *Carthamus tinctorius* | Cumalic acid | 500-05-0 | 43.10 | -0.01 | 0.03 | 140.10 | -0.11 | 1 | 4 |
| **169** | *Carthamus tinctorius* | Nonanal | 124-19-6 | 40.28 | 1.31 | 0.02 | 142.27 | 3.22 | 0 | 1 |
| **170** | *Carthamus tinctorius* | 3-Formylindole | 487-89-8 | 19.82 | 1.25 | 0.04 | 145.17 | 1.88 | 1 | 1 |
| **171** | *Carthamus tinctorius* | L-Lysine | 6899-06-5 | 29.33 | -0.66 | 0.02 | 146.22 | -0.68 | 5 | 4 |
| **172** | *Carthamus tinctorius* | Triethyl orthoformate | 122-51-0 | 40.81 | 0.96 | 0.02 | 148.23 | 1.61 | 0 | 3 |
| **173** | *Carthamus tinctorius* | D-(-)-Arabinose | 147-81-9 | 1.87 | -1.59 | 0.02 | 150.15 | -2.17 | 4 | 5 |
| **174** | *Carthamus tinctorius* | 3-phenylpropionic acid | 501-52-0 | 35.83 | 0.92 | 0.03 | 150.19 | 1.93 | 1 | 2 |
| **175** | *Carthamus tinctorius* | Levoverbenone | 1196-01-6 | 50.66 | 1.26 | 0.06 | 150.24 | 1.94 | 0 | 1 |
| **176** | *Carthamus tinctorius* | trans-2,4-Decadienal | 2363-88-4 | 51.03 | 1.40 | 0.02 | 152.26 | 3.21 | 0 | 1 |
| **177** | *Carthamus tinctorius* | (+)-Terpinen-4-ol | 2438-10-0 | 81.41 | 1.36 | 0.03 | 154.28 | 2.55 | 1 | 1 |
| **178** | *Carthamus tinctorius* | Decanal | 112-31-2 | 29.81 | 1.34 | 0.02 | 156.30 | 3.68 | 0 | 1 |
| **179** | *Carthamus tinctorius* | Tridecene-3,5,7,9,11-pentayne | 2060-59-5 | 37.14 | 2.53 | 0.04 | 162.19 | 4.30 | 0 | 0 |
| **180** | *Carthamus tinctorius* | Methyl cinnamate | 103-26-4 | 18.42 | 1.30 | 0.04 | 162.20 | 2.15 | 0 | 2 |
| **181** | *Carthamus tinctorius* | *p*-Hydroxycinnamic acid | 4501-31-9 | 45.98 | 0.46 | 0.04 | 164.17 | 1.64 | 2 | 3 |
| **182** | *Carthamus tinctorius* | (*E*)-1,3-Tridecadiene-5,7,9,11-tetrayne | N/A | 37.30 | 2.49 | 0.04 | 164.21 | 4.18 | 0 | 0 |
| **183** | *Carthamus tinctorius* | 1,11(Z)-Tridecadien-3,5,7,9-tetrayne | 2345-16-6 | 43.51 | 2.40 | 0.04 | 164.21 | 4.18 | 0 | 0 |
| **184** | *Carthamus tinctorius* | (2*R*)-2-Ammonio-3-*p*henylpropanoate | 673-06-3 | 92.86 | 0.18 | 0.04 | 165.21 | 0.96 | 3 | 3 |
| **185** | *Carthamus tinctorius* | (3*E*,5*E*)-Trideca-1,3,5-trien-7,9,11-triyne | N/A | 33.32 | 2.27 | 0.04 | 166.23 | 4.06 | 0 | 0 |
| **186** | *Carthamus tinctorius* | 1,3(*Z*),11(*E*)-Tridecatrien-5,7,9-triyne | 124604-44-0 | 41.57 | 2.29 | 0.04 | 166.23 | 4.06 | 0 | 0 |
| **187** | *Carthamus tinctorius* | 1,3,5,11-Tridecatetraene-7,9-diyne | 17091-00-8 | 36.62 | 2.19 | 0.04 | 168.25 | 3.93 | 0 | 0 |
| **188** | *Carthamus tinctorius* | Decanoic acid | 334-48-5 | 26.74 | 0.96 | 0.03 | 172.30 | 3.63 | 1 | 2 |
| **189** | *Carthamus tinctorius* | Caffeic acid | 501-16-6 | 54.97 | 0.21 | 0.05 | 180.17 | 1.37 | 3 | 4 |
| **190** | *Carthamus tinctorius* | Aldehydo-*D*-galactose | 59-23-4 | 47.81 | -1.67 | 0.03 | 180.18 | -2.68 | 5 | 6 |
| **191** | *Carthamus tinctorius* | (*S*)-4,4,7a-Trimethyl-5,6,7,7a-tetrahydrobenzofuran-2(4H)-one | 81800-41-1 | 36.84 | 1.20 | 0.07 | 180.27 | 2.48 | 0 | 2 |
| **192** | *Carthamus tinctorius* | 3,4,5-Trimethoxytoluene | 6443-69-2 | 23.73 | 1.32 | 0.04 | 182.24 | 2.27 | 0 | 3 |
| **193** | *Carthamus tinctorius* | Cyclododecanol | 1724-39-6 | 41.45 | 1.21 | 0.04 | 184.36 | 4.24 | 1 | 1 |
| **194** | *Carthamus tinctorius* | *β*-Ionone | 79-77-6 | 20.63 | 1.41 | 0.05 | 192.33 | 3.22 | 0 | 1 |
| **195** | *Carthamus tinctorius* | Tetradecene | 1120-36-1 | 6.15 | 1.85 | 0.04 | 196.42 | 6.37 | 0 | 0 |
| **196** | *Carthamus tinctorius* | Lauric acid | 143-07-7 | 23.59 | 1.02 | 0.04 | 200.36 | 4.54 | 1 | 1 |
| **197** | *Carthamus tinctorius* | Fluoranthene | 206-44-0 | 24.70 | 1.97 | 0.18 | 202.26 | 3.95 | 0 | 0 |
| **198** | *Carthamus tinctorius* | (1*S*,4*Z*,9*R*)-4,11,11-trimethyl-8-methylidenebicyclo[7.2.0]undec-4-ene | 10579-93-8 | 27.36 | 1.85 | 0.09 | 204.39 | 4.75 | 0 | 0 |
| **199** | *Carthamus tinctorius* | *α*-humulene | 6753-98-6 | 22.98 | 1.88 | 0.06 | 204.39 | 5.04 | 0 | 0 |
| **200** | *Carthamus tinctorius* | Junipene | 475-20-7 | 44.07 | 1.82 | 0.11 | 204.39 | 4.18 | 0 | 0 |
| **201** | *Carthamus tinctorius* | *β*-Caryophyllene | 947-59-1 | 29.59 | 1.84 | 0.12 | 204.39 | 4.17 | 0 | 0 |
| **202** | *Carthamus tinctorius* | *β*-Caryophyllene | 87-44-5 | 29.70 | 1.83 | 0.09 | 204.39 | 4.75 | 0 | 0 |
| **203** | *Carthamus tinctorius* | (*E*)-*β*-Farnesene | 502-60-3 | 17.42 | 1.95 | 0.05 | 204.39 | 5.52 | 0 | 0 |
| **204** | *Carthamus tinctorius* | (-)-*α*-Cedrene | 69-61-4 | 55.56 | 1.81 | 0.10 | 204.39 | 4.12 | 0 | 0 |
| **205** | *Carthamus tinctorius* | 2,4-Di-tert-butylphenol | 96-76-4 | 26.74 | 1.68 | 0.06 | 206.36 | 4.36 | 1 | 1 |
| **206** | *Carthamus tinctorius* | 4-[(*Z*)-3-Hydroxyprop-1-enyl]-2,6-dimethoxyphenol | 118-34-3 | 49.15 | 0.56 | 0.06 | 210.25 | 1.39 | 2 | 4 |
| **207** | *Carthamus tinctorius* | 1-Pentadecene | 27251-68-9 | 17.72 | 1.84 | 0.05 | 210.45 | 6.82 | 0 | 0 |
| **208** | *Carthamus tinctorius* | (2*S*)-2,3-Dihydroxypropyl octanoate | 68132-29-6 | 22.28 | 0.06 | 0.05 | 218.33 | 1.92 | 2 | 4 |
| **209** | *Carthamus tinctorius* | Cincofarm | 4350-09-8 | 74.63 | -0.15 | 0.10 | 220.25 | 0.98 | 5 | 4 |
| **210** | *Carthamus tinctorius* | (*Z*)-2-methyl-5-[(1*R*,2*S*,4*S*)-2-methyl-3-methylene-2-norbornanyl]pent-2-en-1-ol | 115-71-9 | 62.85 | 1.26 | 0.09 | 220.39 | 3.66 | 1 | 1 |
| **211** | *Carthamus tinctorius* | (-)-Caryophyllene oxide | 1139-30-6 | 32.67 | 1.58 | 0.13 | 220.39 | 3.52 | 0 | 1 |
| **212** | *Carthamus tinctorius* | Vomifoliol | 23526-45-6 | 29.01 | -0.08 | 0.08 | 224.33 | 1.25 | 2 | 3 |
| **213** | *Carthamus tinctorius* | Hexadecene | 629-73-2 | 5.34 | 1.83 | 0.06 | 224.48 | 7.28 | 0 | 0 |
| **214** | *Carthamus tinctorius* | Tetradecanoic acid | 544-63-8 | 21.18 | 1.07 | 0.07 | 228.42 | 5.46 | 1 | 2 |
| **215** | *Carthamus tinctorius* | (*E*,*Z*)-2,8-Decadien-4,6-diyn-1-yl 3-methylbutanoate | N/A | 26.60 | 1.65 | 0.08 | 230.33 | 4.06 | 0 | 2 |
| **216** | *Carthamus tinctorius* | [(*Z*)-dec-8-en-4,6-diynyl] 3-methylbutanoate | N/A | 27.82 | 1.56 | 0.08 | 232.35 | 4.37 | 0 | 2 |
| **217** | *Carthamus tinctorius* | (8*Z*,11*Z*,14*Z*)-Heptadeca-1,8,11,14-tetraene | 10482-53-8 | 44.06 | 1.87 | 0.08 | 232.45 | 6.40 | 0 | 0 |
| **218** | *Carthamus tinctorius* | Deca-4,6-diyn-1-yl 3-methylbutanoate | N/A | 20.69 | 1.59 | 0.08 | 234.37 | 4.82 | 0 | 2 |
| **219** | *Carthamus tinctorius* | Diamylphenol | 138-00-1 | 17.62 | 1.73 | 0.08 | 234.42 | 6.19 | 1 | 1 |
| **220** | *Carthamus tinctorius* | Dihydroaplotaxene | 56134-03-3 | 4.78 | 1.90 | 0.08 | 234.47 | 6.85 | 0 | 0 |
| **221** | *Carthamus tinctorius* | Heptadecene | 6765-39-5 | 20.03 | 1.84 | 0.08 | 238.51 | 7.74 | 0 | 0 |
| **222** | *Carthamus tinctorius* | 7,8-Dimethyl-1H-pyrimido[5,6-g]quinoxaline-2,4-dione | N/A | 45.75 | 0.06 | 0.19 | 242.26 | 0.59 | 2 | 6 |
| **223** | *Carthamus tinctorius* | Pentadecylic acid | 1002-84-2 | 20.18 | 1.08 | 0.08 | 242.45 | 5.91 | 1 | 2 |
| **224** | *Carthamus tinctorius* | Uridine | 26287-69-4 | 23.40 | -1.07 | 0.11 | 244.23 | -2.45 | 4 | 8 |
| **225** | *Carthamus tinctorius* | *α*-Terthiophene | 1081-34-1 | 19.56 | 1.46 | 0.07 | 248.1 | 5.02 | 0 | 0 |
| **226** | *Carthamus tinctorius* | Octadecene | 112-88-9 | 19.21 | 1.88 | 0.09 | 252.54 | 8.19 | 0 | 0 |
| **227** | *Carthamus tinctorius* | Palmitoleic acid | 373-49-9 | 35.78 | 1.18 | 0.10 | 254.46 | 5.92 | 1 | 2 |
| **228** | *Carthamus tinctorius* | Palmitic acid | 57-10-3 | 19.30 | 1.09 | 0.10 | 256.48 | 6.37 | 1 | 2 |
| **229** | *Carthamus tinctorius* | Adenosine | 30143-02-3 | 15.98 | -1.56 | 0.18 | 267.28 | -2.02 | 5 | 8 |
| **230** | *Carthamus tinctorius* | Baicalein | 491-67-8 | 33.52 | 0.63 | 0.21 | 270.25 | 2.33 | 3 | 5 |
| **231** | *Carthamus tinctorius* | Apigenin | 520-36-5 | 23.06 | 0.43 | 0.21 | 270.25 | 2.33 | 2 | 5 |
| **232** | *Carthamus tinctorius* | Benzyl glucopyranoside | 4304-12-5 | 12.39 | -0.70 | 0.14 | 270.31 | -0.52 | 4 | 6 |
| **233** | *Carthamus tinctorius* | Daturic acid | 506-12-7 | 18.51 | 1.12 | 0.12 | 270.51 | 6.82 | 1 | 2 |
| **234** | *Carthamus tinctorius* | (2*R*,6*R*,10*R*)-6,10,14-trimethylpentadecan-2-ol | N/A | 20.94 | 1.33 | 0.10 | 270.56 | 6.67 | 1 | 1 |
| **235** | *Carthamus tinctorius* | *γ*-Linolenic acid | 506-26-3 | 45.01 | 1.20 | 0.15 | 278.48 | 5.95 | 1 | 2 |
| **236** | *Carthamus tinctorius* | Linolenic acid | 60-33-3 | 45.01 | 1.21 | 0.15 | 278.48 | 5.95 | 1 | 2 |
| **237** | *Carthamus tinctorius* | Linoleic acid | 60-33-3 | 41.90 | 1.16 | 0.14 | 280.50 | 6.39 | 1 | 2 |
| **238** | *Carthamus tinctorius* | Oleic acid | 8046-01-3 | 33.13 | 1.17 | 0.14 | 282.52 | 6.84 | 1 | 2 |
| **239** | *Carthamus tinctorius* | Guanosine | 118-00-3 | 21.43 | -1.21 | 0.21 | 283.28 | -2.41 | 6 | 9 |
| **240** | *Carthamus tinctorius* | Acacetin | 480-44-4 | 34.97 | 0.67 | 0.24 | 284.28 | 2.59 | 2 | 5 |
| **241** | *Carthamus tinctorius* | Stearic acid | 57-11-4 | 17.83 | 1.15 | 0.14 | 284.54 | 7.28 | 1 | 2 |
| **242** | *Carthamus tinctorius* | Carthamone_qt | N/A | 51.03 | -0.31 | 0.20 | 286.25 | 0.70 | 3 | 6 |
| **243** | *Carthamus tinctorius* | Scutellarein | 529-53-3 | 18.97 | 0.31 | 0.24 | 286.25 | 2.07 | 4 | 6 |
| **244** | *Carthamus tinctorius* | Kaempferol | 520-18-3 | 67.43 | 0.26 | 0.24 | 286.25 | 1.77 | 4 | 6 |
| **245** | *Carthamus tinctorius* | Luteolin | 491-70-3 | 36.16 | 0.19 | 0.25 | 286.25 | 2.07 | 4 | 6 |
| **246** | *Carthamus tinctorius* | Hydroxysafflor yellow A_qt | N/A | 13.02 | -0.43 | 0.20 | 288.27 | 0.45 | 4 | 6 |
| **247** | *Carthamus tinctorius* | 6-Hydroxynaringenin | 479-54-9 | 33.23 | 0.27 | 0.24 | 288.27 | 2.03 | 4 | 6 |
| **248** | *Carthamus tinctorius* | Safflomin A_qt | N/A | 5.85 | -0.50 | 0.20 | 288.27 | 0.45 | 4 | 6 |
| **249** | *Carthamus tinctorius* | Eriodictyol | 552-58-9 | 71.79 | 0.17 | 0.24 | 288.27 | 2.03 | 4 | 6 |
| **250** | *Carthamus tinctorius* | Methyl linolenate | 301-00-8 | 46.15 | 1.48 | 0.17 | 292.51 | 6.20 | 0 | 2 |
| **251** | *Carthamus tinctorius* | Phytol | 150-86-7 | 33.82 | 1.23 | 0.13 | 296.60 | 7.34 | 1 | 1 |
| **252** | *Carthamus tinctorius* | 6-Hydroxykaempferol | 4324-55-4 | 62.13 | 0.16 | 0.27 | 302.25 | 1.50 | 5 | 7 |
| **253** | *Carthamus tinctorius* | Quercetin | 117-39-5 | 46.43 | 0.05 | 0.28 | 302.25 | 1.50 | 5 | 7 |
| **254** | *Carthamus tinctorius* | *N*-Docosane | 629-97-0 | 8.37 | 1.85 | 0.18 | 310.68 | 10.41 | 0 | 0 |
| **255** | *Carthamus tinctorius* | Arachic acid | 506-30-9 | 16.66 | 1.18 | 0.19 | 312.60 | 8.19 | 1 | 2 |
| **256** | *Carthamus tinctorius* | Precarthamin_qt | N/A | 17.48 | -0.02 | 0.24 | 316.38 | 3.17 | 5 | 5 |
| **257** | *Carthamus tinctorius* | Myricetin | 529-44-2 | 13.75 | -0.15 | 0.31 | 318.25 | 1.24 | 6 | 8 |
| **258** | *Carthamus tinctorius* | quercetagetin | 90-18-6 | 45.01 | -0.06 | 0.31 | 318.25 | 1.24 | 6 | 8 |
| **259** | *Carthamus tinctorius* | L-*α*-Palmitin | 19670-51-0 | 26.66 | 0.30 | 0.22 | 330.57 | 5.57 | 2 | 4 |
| **260** | *Carthamus tinctorius* | propanetriol-a-arabinofuranosyl(1→4) | N/A | 0.87 | -2.72 | 0.24 | 342.34 | -4.33 | 8 | 11 |
| **261** | *Carthamus tinctorius* | N-Pentacosane | 629-99-2 | 8.25 | 1.90 | 0.27 | 352.77 | 11.78 | 0 | 0 |
| **262** | *Carthamus tinctorius* | 3-Caffeoylquinic acid | 327-97-9 | 11.93 | -1.03 | 0.33 | 354.34 | -0.42 | 6 | 9 |
| **263** | *Carthamus tinctorius* | Terephthaldehyde | 623-27-8 | 11.93 | -1.35 | 0.33 | 354.34 | -0.42 | 6 | 9 |
| **264** | *Carthamus tinctorius* | 4-[(*E*)-4-(3,5-dimethoxy-4-oxo-1-cyclohexa-2,5-dienylidene)but-2-enylidene]-2,6-dimethoxycyclohexa-2,5-dine-1-one | N/A | 48.47 | 0.81 | 0.36 | 356.40 | 0.36 | 0 | 6 |
| **265** | *Carthamus tinctorius* | Syrigin | 118-34-3 | 14.64 | -1.01 | 0.32 | 372.41 | -0.51 | 5 | 9 |
| **266** | *Carthamus tinctorius* | Pyrethrin II | 121-29-9 | 48.36 | 0.53 | 0.35 | 372.50 | 3.74 | 0 | 5 |
| **267** | *Carthamus tinctorius* | Vitamin-G | 83-88-5 | 6.79 | -1.22 | 0.50 | 376.41 | 0.23 | 5 | 10 |
| **268** | *Carthamus tinctorius* | Corchoionoside C | 54835-70-0 | 10.81 | -1.25 | 0.36 | 386.49 | -0.50 | 5 | 8 |
| **269** | *Carthamus tinctorius* | Cholesterol | 57-88-5 | 37.87 | 1.43 | 0.68 | 386.73 | 7.38 | 1 | 1 |
| **270** | *Carthamus tinctorius* | Octacosane | 630-02-4 | 8.15 | 1.91 | 0.37 | 394.86 | 13.15 | 0 | 0 |
| **271** | *Carthamus tinctorius* | Lirioresinol-A | 6216-82-6P | 1.76 | 0.41 | 0.67 | 404.45 | 1.85 | 3 | 8 |
| **272** | *Carthamus tinctorius* | Nonacosane | 630-03-5 | 8.12 | 1.92 | 0.39 | 408.89 | 13.60 | 0 | 0 |
| **273** | *Carthamus tinctorius* | *γ*-Tocotrienol | 14101-61-2 | 20.30 | 1.55 | 0.53 | 410.70 | 9.18 | 1 | 2 |
| **274** | *Carthamus tinctorius* | Stigmasterol | 83-48-7 | 43.83 | 1.44 | 0.76 | 412.77 | 7.64 | 1 | 1 |
| **275** | *Carthamus tinctorius* | Clionasterol | 83-46-5 | 36.91 | 1.45 | 0.75 | 414.79 | 8.08 | 1 | 1 |
| **276** | *Carthamus tinctorius* | *β*-sitosterol | 83-46-5 | 36.91 | 1.32 | 0.75 | 414.79 | 8.08 | 1 | 1 |
| **277** | *Carthamus tinctorius* | *β*-Tocopherol | 1406-18-4 | 15.62 | 1.67 | 0.52 | 416.76 | 9.93 | 1 | 2 |
| **278** | *Carthamus tinctorius* | (+)-Syringaresinol | 21453-69-0 | 3.29 | 0.47 | 0.72 | 418.48 | 2.10 | 2 | 8 |
| **279** | *Carthamus tinctorius* | Nonacosanol | 6624-76-6 | 10.57 | 1.48 | 0.43 | 424.89 | 12.38 | 1 | 1 |
| **280** | *Carthamus tinctorius* | Lupeol | 545-47-1 | 12.12 | 1.46 | 0.78 | 426.80 | 7.40 | 1 | 1 |
| **281** | *Carthamus tinctorius* | Vitamin E | 59-02-9 | 14.26 | 1.70 | 0.55 | 430.79 | 10.42 | 1 | 2 |
| **282** | *Carthamus tinctorius* | Kaempferin | 482-39-3 | 3.83 | -0.77 | 0.70 | 432.41 | 0.57 | 6 | 10 |
| **283** | *Carthamus tinctorius* | Kaempferol-7-O-rhamnoside | 20196-89-8 | 9.44 | -0.83 | 0.72 | 432.41 | 0.76 | 6 | 10 |
| **284** | *Carthamus tinctorius* | Baicalin | 31564-28-0 | 40.12 | -0.85 | 0.75 | 446.39 | 0.64 | 6 | 11 |
| **285** | *Carthamus tinctorius* | 5,7-Dihydroxy-2-(4-hydroxyphenyl)-3-[(2*S*,3*S*,4*S*,5*S*,6*R*)-3,4,5-trihydroxy-6-(hydroxymethyl)oxan-2-yl]oxychromen-4-one | N/A | 3.32 | -1.36 | 0.74 | 448.41 | -0.32 | 7 | 11 |
| **286** | *Carthamus tinctorius* | Safflow yellow A_qt | N/A | 16.07 | -2.14 | 0.79 | 448.41 | -1.95 | 7 | 11 |
| **287** | *Carthamus tinctorius* | Kaempferol-3-O-galactoside | 23627-87-4 | 3.10 | -1.17 | 0.74 | 448.41 | -0.32 | 7 | 11 |
| **288** | *Carthamus tinctorius* | Carthamone | 86579-00-2 | 5.93 | -1.81 | 0.63 | 448.41 | -1.39 | 6 | 11 |
| **289** | *Carthamus tinctorius* | Astragalin | 480-10-4 | 14.03 | -1.34 | 0.74 | 448.41 | -0.32 | 7 | 11 |
| **290** | *Carthamus tinctorius* | Neocarthamin | 519-54-0 | 17.81 | -1.27 | 0.77 | 450.43 | 0.13 | 7 | 11 |
| **291** | *Carthamus tinctorius* | *N*-Dotriacontane | 544-85-4 | 8.03 | 1.96 | 0.46 | 450.98 | 14.97 | 0 | 0 |
| **292** | *Carthamus tinctorius* | Safflomin-C_qt | N/A | 3.53 | -0.85 | 0.59 | 452.44 | 1.52 | 6 | 9 |
| **293** | *Carthamus tinctorius* | Hydroxybenzoyl-counmaricanhydride | N/A | 1.68 | -0.46 | 0.78 | 456.42 | 4.17 | 3 | 8 |
| **304** | *Carthamus tinctorius* | Lignan | N/A | 43.32 | 0.42 | 0.65 | 458.55 | 3.78 | 0 | 8 |
| **305** | *Carthamus tinctorius* | Scutellarin | 1329-06-2 | 2.64 | -1.08 | 0.79 | 462.39 | 0.37 | 7 | 12 |
| **306** | *Carthamus tinctorius* | Quercimeritrin | 491-50-9 | 2.85 | -1.36 | 0.79 | 464.41 | -0.40 | 8 | 12 |
| **307** | *Carthamus tinctorius* | 6-Hydroxykaempferol-3-*O*-*β*-*D*-glucoside | 145134-61-8 | 1.85 | -1.52 | 0.76 | 464.41 | -0.59 | 8 | 12 |
| **298** | *Carthamus tinctorius* | 6-Hydroxykaempferol-7-*O*-*β*-*D*-glucoside | N/A | 2.87 | -1.47 | 0.78 | 464.41 | -0.40 | 8 | 12 |
| **299** | *Carthamus tinctorius* | Isoquercitrin | 482-35-9 | 1.86 | -1.66 | 0.77 | 464.41 | -0.59 | 8 | 12 |
| **300** | *Carthamus tinctorius* | Quercimeritrin | 491-50-9 | 2.85 | -1.36 | 0.79 | 464.41 | -0.40 | 8 | 12 |
| **301** | *Carthamus tinctorius* | Spiraeoside | 20229-56-5 | 3.52 | -1.53 | 0.81 | 464.41 | -0.40 | 8 | 12 |
| **302** | *Carthamus tinctorius* | Dotriacontanol | 6624-79-9 | 10.25 | 1.48 | 0.46 | 466.98 | 13.74 | 1 | 1 |
| **303** | *Carthamus tinctorius* | *β*-amyrin acetate | 1616-93-9 | 9.11 | 1.42 | 0.74 | 468.84 | 7.68 | 0 | 2 |
| **304** | *Carthamus tinctorius* | hentriacontane-8,10-diol | N/A | 13.41 | 0.81 | 0.50 | 468.95 | 11.92 | 2 | 2 |
| **305** | *Carthamus tinctorius* | Quercetin-6-glucoside | N/A | 16.75 | -1.58 | 0.80 | 480.41 | -0.67 | 9 | 13 |
| **306** | *Carthamus tinctorius* | Tagetiin | 60671-81-0 | 28.34 | -1.41 | 0.78 | 480.41 | -0.85 | 9 | 13 |
| **307** | *Carthamus tinctorius* | *β*-carotene | 116-32-5 | 37.18 | 2.25 | 0.58 | 536.96 | 12.00 | 0 | 0 |
| **308** | *Carthamus tinctorius* | Phytofluene | 540-05-6 | 43.18 | 2.29 | 0.50 | 543.02 | 14.10 | 0 | 0 |
| **309** | *Carthamus tinctorius* | Phytoene | 540-04-5 | 39.56 | 2.22 | 0.50 | 545.04 | 14.54 | 0 | 0 |
| **310** | *Carthamus tinctorius* | Lutein | 127-40-2 | 15.74 | 1.12 | 0.54 | 568.96 | 9.47 | 2 | 2 |
| **311** | *Carthamus tinctorius* | 1,3-Dipalmitin | 502-52-3 | 21.16 | 0.39 | 0.44 | 569.03 | 12.55 | 1 | 5 |
| **312** | *Carthamus tinctorius* | L-1,2-Dipalmitin | N/A | 21.28 | 0.38 | 0.49 | 569.03 | 12.55 | 1 | 5 |
| **313** | *Carthamus tinctorius* | Sitogluside | 474-58-8 | 20.63 | -0.14 | 0.62 | 576.95 | 6.34 | 4 | 6 |
| **314** | *Carthamus tinctorius* | Luteolin-7-O-*β*-D-glucoside | 5373-11-5 | 3.72 | -2.10 | 0.76 | 580.54 | -1.25 | 9 | 15 |
| **315** | *Carthamus tinctorius* | Tricoumaroyl spermidine | N/A | 1.45 | -0.12 | 0.53 | 583.74 | 4.26 | 5 | 9 |
| **316** | *Carthamus tinctorius* | N1,N5-(*Z*)-N10-(*E*)-tri-*p*-coumaroylspermidine | N/A | 1.45 | -0.14 | 0.53 | 583.74 | 4.26 | 5 | 9 |
| **317** | *Carthamus tinctorius* | Flavoxanthin | 512-29-8 | 60.41 | 0.97 | 0.56 | 584.96 | 8.24 | 2 | 3 |
| **318** | *Carthamus tinctorius* | Nicotiflorin | 17650-84-9 | 3.64 | -1.77 | 0.73 | 594.57 | -1.18 | 9 | 15 |
| **319** | *Carthamus tinctorius* | Safflor yellow A | 85532-77-0 | 22.75 | -2.52 | 0.75 | 594.57 | -4.03 | 10 | 15 |
| **320** | *Carthamus tinctorius* | Tinctormine | 149475-43-4 | 18.09 | -3.15 | 0.68 | 595.56 | -4.33 | 13 | 16 |
| **321** | *Carthamus tinctorius* | Sophoraflavonoloside | 19895-95-5 | 5.30 | -2.42 | 0.71 | 610.57 | -2.07 | 10 | 16 |
| **322** | *Carthamus tinctorius* | Kaempferol-3-O-*β*-D-rutinoside | 17650-84-9 | 5.51 | -2.59 | 0.65 | 610.57 | -2.24 | 10 | 16 |
| **323** | *Carthamus tinctorius* | Rutin | 153-18-4 | 11.70 | -1.93 | 0.68 | 610.57 | -1.45 | 10 | 16 |
| **324** | *Carthamus tinctorius* | Quercetin-3-rhamnoside-7-glucoside | 17306-45-5 | 5.51 | -2.21 | 0.71 | 610.57 | -1.60 | 10 | 16 |
| **325** | *Carthamus tinctorius* | Hydroxysafflor yllow A | 78281-02-4 | 4.77 | -2.77 | 0.68 | 612.59 | -4.45 | 12 | 16 |
| **326** | *Carthamus tinctorius* | Safflomin A | 873799-90-7 | 3.53 | -3.03 | 0.68 | 612.59 | -4.45 | 12 | 16 |
| **327** | *Carthamus tinctorius* | Safflomin C | 873799-91-8 | 5.57 | -2.01 | 0.66 | 614.60 | -0.95 | 10 | 14 |
| **328** | *Carthamus tinctorius* | Carthamin_qt | N/A | 6.75 | -2.35 | 0.60 | 620.55 | 0.07 | 9 | 14 |
| **329** | *Carthamus tinctorius* | Kaempferol-3-O-rutinoside-7-O-galactoside_qt | N/A | 14.28 | -2.00 | 0.63 | 624.60 | -1.99 | 9 | 16 |
| **330** | *Carthamus tinctorius* | Sesquiterpene | N/A | 5.86 | -2.93 | 0.67 | 626.57 | -2.49 | 11 | 17 |
| **331** | *Carthamus tinctorius* | 6-Hydroxykaempferol-3,6-di-O-D-glucoside | N/A | 4.21 | -2.68 | 0.68 | 626.57 | -2.49 | 11 | 17 |
| **332** | *Carthamus tinctorius* | 6-Hydroxykaempferol-6,7-di-O-D-glucoside | 205527-00-0 | 12.77 | -2.49 | 0.69 | 626.57 | -2.31 | 11 | 17 |
| **333** | *Carthamus tinctorius* | Quercetin-3,7-di-O-*β*-D-glucoside | 56782-99-1 | 5.86 | -2.57 | 0.67 | 626.57 | -2.49 | 11 | 17 |
| **334** | *Carthamus tinctorius* | Precarthamin_qt | N/A | 6.83 | -2.04 | 0.55 | 660.62 | 0.54 | 9 | 14 |
| **335** | *Carthamus tinctorius* | Glyceryl pps | 65236-61-5 | 29.61 | 0.45 | 0.35 | 663.15 | 14.49 | 0 | 6 |
| **336** | *Carthamus tinctorius* | Lupeol palmitate | 32214-80-5 | 33.98 | 1.52 | 0.32 | 673.89 | 14.38 | 0 | 2 |
| **337** | *Carthamus tinctorius* | Phospholipid | 131933-70-5 | 15.43 | -0.72 | 0.45 | 673.89 | 4.72 | 2 | 13 |
| **338** | *Carthamus tinctorius* | Thymopentin | 69558-55-0 | 1.24 | -1.91 | 0.46 | 679.88 | -1.32 | 15 | 18 |
| **339** | *Carthamus tinctorius* | Safflower yellow B_qt | N/A | 9.47 | -3.16 | 0.41 | 766.76 | -1.57 | 13 | 17 |
| **340** | *Carthamus tinctorius* | Kaempferol-3-O-rutinoside-7-O-galactoside | N/A | 3.05 | -3.39 | 0.34 | 772.73 | -4.14 | 13 | 21 |
| **341** | *Carthamus tinctorius* | 6-Hydroxykaempferol-3,6,7-tri-O-*β*-D-glucoside | N/A | 3.14 | -3.91 | 0.39 | 788.73 | -4.40 | 14 | 22 |
| **342** | *Carthamus tinctorius* | 6-Hydroxykaempferol-3-O-*β*-D-Rutoside-6-O-*β*-D-glucoside | N/A | 3.02 | -3.73 | 0.34 | 788.73 | -4.41 | 14 | 22 |
| **343** | *Carthamus tinctorius* | Sitoindoside II | 53657-29-7 | 26.94 | 0.12 | 0.16 | 841.45 | 13.78 | 3 | 7 |
| **344** | *Carthamus tinctorius* | Carthamin | 36338-96-2 | 3.09 | -4.28 | 0.25 | 910.85 | -4.13 | 15 | 22 |
| **345** | *Carthamus tinctorius* | Precarthamin | 168216-23-7 | 3.01 | -4.84 | 0.21 | 956.84 | -4.69 | 17 | 24 |
| **346** | *Carthamus tinctorius* | Safflor yellow B | 91574-92-4 | 3.01 | -4.20 | 0.17 | 1062.07 | -6.80 | 21 | 27 |

Danggui: the radix of *Angelica sinensis*, Honghua: the florets of *Carthamus tinctorius*, OB: oral bioavailability, DL: druglikeness, MW: molecular weight, nHDon: number of donor atoms for H-bonds, nHAcc: number of acceptor atoms for H-bonds and MLogP: Moriguchi octanol-water partition coeff.(LogP).

qt represents the molecule with deglycosylation.

**Table S2**. Key parameters of C-T, T-D and T-P networks.

| Parameters | C-T | T-D | T-P |
| --- | --- | --- | --- |
| Number of nodes | 73 | 49 | 62 |
| Network density | 0.090 | 0.066 | 0.061 |
| Network heterogeneity | 0.515 | 1.638 | 0.757 |
| Average number of neighbors | 6.466 | 3.184 | 3.710 |
| Characteristic path length | 2.689 | 2.435 | 3.748 |
| Shortest paths | 5256 (100%) | 2352 (100%) | 3782 (100%) |
| Network centralization | 0.150 | 0.713 | 0.123 |


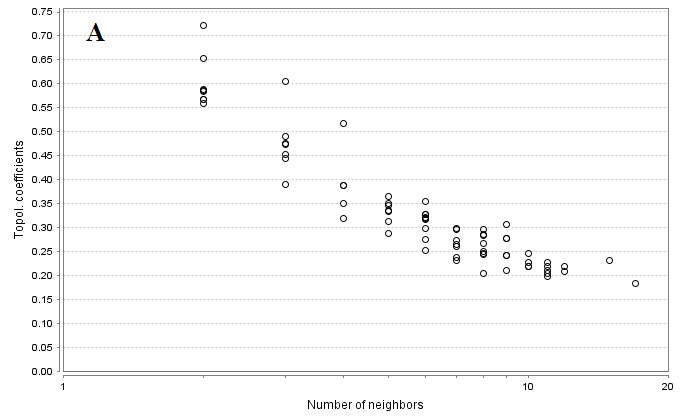

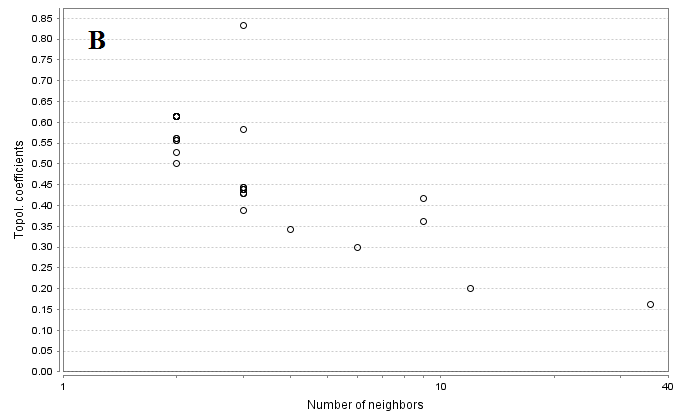

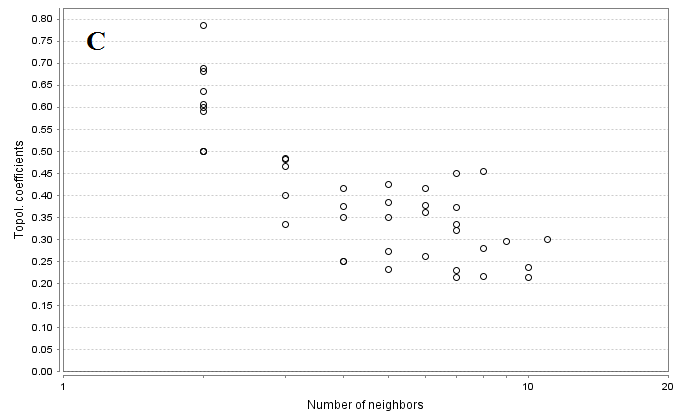


**Figure S1**. Topological coefficients of C-T (A), T-D (B) and T-P (C) networks.

**Table S3**. The number of targets, degrees, and references, network based efficacy (NE), and contribution indexes (CI) of active ingredients in Danggui-Honghua.

| No. | No. targets | No. degrees | No. references | NE | CI | No. | No. targets | No. degrees | No. references | NE | CI |
| --- | --- | --- | --- | --- | --- | --- | --- | --- | --- | --- | --- |
| HH-1 | 7 | 26 | 0 | 0 | 0 | DG-1 | 15 | 30 | 37 | 1110 | 27.23 |
| HH-2 | 5 | 26 | 4 | 104 | 2.55 | DG-2 | 8 | 22 | 0 | 0 | 0 |
| HH-3 | 17 | 29 | 42 | 1218 | 29.87 | DG-3 | 9 | 26 | 1 | 26 | 0.64 |
| HH-4 | 3 | 22 | 1 | 22 | 0.54 | DG-4 | 5 | 25 | 0 | 0 | 0 |
| HH-5 | 5 | 20 | 11 | 220 | 5.40 | DG-5 | 10 | 26 | 15 | 390 | 9.57 |
| HH-6 | 8 | 29 | 0 | 0 | 0 | DG-6 | 4 | 16 | 0 | 0 | 0 |
| HH-7 | 10 | 24 | 2 | 48 | 1.18 | DG-7 | 6 | 22 | 0 | 0 | 0 |
| HH-8 | 6 | 27 | 2 | 54 | 1.32 | DG-8 | 4 | 21 | 0 | 0 | 0 |
| HH-9 | 12 | 23 | 2 | 46 | 1.13 | DG-9 | 6 | 25 | 2 | 50 | 1.23 |
| HH-10 | 5 | 22 | 2 | 44 | 1.08 | DG-10 | 3 | 20 | 0 | 0 | 0 |
| HH-11 | 8 | 25 | 0 | 0 | 0 | DG-11 | 7 | 28 | 1 | 28 | 0.69 |
| HH-12 | 9 | 22 | 1 | 22 | 0.54 | DG-12 | 11 | 29 | 19 | 551 | 13.51 |
| HH-13 | 5 | 22 | 0 | 0 | 0 | DG-13 | 4 | 16 | 0 | 0 | 0 |
| HH-14 | 10 | 22 | 1 | 22 | 0.54 | DG-14 | 6 | 22 | 3 | 66 | 1.62 |
| HH-15 | 11 | 28 | 2 | 56 | 1.37 | DG-15 | 9 | 23 | 0 | 0 | 0 |
| HH-16 | 8 | 27 | 0 | 0 | 0 |  |  |  |  |  |  |

No. targets: the number of targets associated with the corresponding ingredient, No. degrees: the degree of target associated with the corresponding ingredient, No. references: the number of blood stasis syndrome-related literature of the corresponding ingredient.
